# Mechanical stimulation of Schwann cells promote peripheral nerve regeneration via extracellular vesicle-mediated transfer of microRNA 23b-3p

**DOI:** 10.7150/thno.44912

**Published:** 2020-07-11

**Authors:** Bing Xia, Jianbo Gao, Shengyou Li, Liangliang Huang, Lei Zhu, Teng Ma, Laihe Zhao, Yujie Yang, Kai Luo, Xiaowei Shi, Liangwei Mei, Hao Zhang, Yi Zheng, Lei Lu, Zhuojing Luo, Jinghui Huang

**Affiliations:** 1 Department of Orthopedics, Xijing Hospital, the Fourth Military Medical University, Xi'an, 710032, People's Republic of China.; 2 Department of Orthopedics, the General Hospital of Central Theater Command of People's Liberation Army, Wuhan, 430070, People's Republic of China.; 3 Department of Spine Surgery, Honghui Hospital Affiliated to Medical School of Xi'an Jiaotong University, Xi'an Shaanxi, 710054, People's Republic of China.; 4 Department of Orthopedics, the 985th Hospital People's Liberation Army Joint Logistics Support Force, Taiyuan, 030000, People's Republic of China.; 5 Department of Spinal Surgery, the People's Hospital of Longhua District, Shenzhen, 518109, People's Republic of China.; 6 Department of Oral Anatomy and Physiology, State Key Laboratory of Military Stomatology & National Clinical Research Center for Oral Diseases & Shaanxi Key Laboratory of Stomatology, School of Stomatology, the Fourth Military Medical University, Xi'an, 710032, People's Republic of China.

**Keywords:** Schwann cell, mechanical stimuli, extracellular vesicle, nerve regeneration, miRNA 23b-3p

## Abstract

**Rationale:** Peripheral nerves are unique in their remarkable elasticity. Schwann cells (SCs), important components of the peripheral nervous system (PNS), are constantly subjected to physiological and mechanical stresses from dynamic stretching and compression forces during movement. So far, it is not clear if SCs sense and respond to mechanical signals. It is also unknown whether mechanical stimuli can interfere with the intercellular communications between neurons and SCs, and what role extracellular vesicles (EVs) play in this process. The present study aimed to examine the effect of mechanical stimuli on the EV-mediated intercellular communication between neurons and SCs, explore their effect on axonal regeneration, and investigate the underlying mechanism.

**Methods:** Purified SCs were stimulated using a magnetic force-based mechanical stimulation (MS) system and EVs were purified from mechanically stimulated SCs (MS-SCs-EVs) and non-stimulated SCs (SCs-EVs). The effect of MS-SCs-EVs on axonal elongation was examined *in vitro* and *in vivo*. High throughput miRNA sequencing was performed to compare the differential miRNA profiles between MS-SCs-EVs and SCs-EVs. The functional role of differentially expressed miRNAs on neurite extension in MS-SCs-EVs was examined. Also, the putative target genes of differentially expressed miRNAs in MS-SCs-EVs were predicted by bioinformatics tools, and the regulatory effect of those miRNAs on putative target genes was validated both* in vitro* and* in vivo*.

**Results:** The MS-SCs-EVs showed an average size of 137.52±1.77 nm, and could be internalized by dorsal root ganglion (DRG) neurons. Compared to SCs-EVs, MS-SCs-EVs showed a stronger ability to enhance neurite outgrowth* in vitro* and nerve regeneration* in vivo*. High throughput miRNA sequencing identified a number of differentially expressed miRNAs in MS-SCs-EVs. Further analysis of those EV-miRNAs demonstrated that miR-23b-3p played a predominant role in MS-SCs-EVs since its deprivation abolished their enhanced axonal elongation. Furthermore, we identified neuropilin 1 (Nrp1) in neurons as the target gene of miR-23b-3p in MS-SCs-EVs. This observation was supported by the evidence that miR-23b-3p could decrease Nrp1-3'-UTR-WT luciferase activity *in vitro* and down-regulate Nrp1 expression in neurons.

**Conclusion:** Our findings suggested that mechanical stimuli are capable of modulating the intercellular communication between neurons and SCs by altering miRNA composition in MS-SCs-EVs. Transfer of miR-23b-3p by MS-SCs-EVs from mechanically stimulated SCs to neurons decreased neuronal Nrp1 expression, which was responsible, at least in part, for the beneficial effect of MS-SCs-EVs on axonal regeneration. Our results highlighted the potential therapeutic value of MS-SCs-EVs and miR-23b-3p-enriched EVs in peripheral nerve injury repair.

## Introduction

Peripheral nerve injury is one of the most frequently encountered clinical settings resulting in morbidity and lifelong disability and poses a considerable burden for both the individual and the society 1, 2. Despite the progress in surgical intervention, the outcome of peripheral nerve injury remains unsatisfactory 3, 4. Schwann cells (SCs), the glial cells in the PNS, form the myelin sheath around neuronal axons, are essential for the rapid saltatory propagation of action potential, and maintain the integrity of axons 5, 6. Upon injury, SCs are reprogrammed to a specialized repair-promoting phenotype, which provides biochemical signals and spatial cues to promote survival of injured neurons, axonal regeneration and reinnervation of target organs 7. Understandably, SCs have been the subject of intensive investigation because of their pivotal role in nerve injury repair, and extensive efforts have been made to promote nerve regeneration by modulating the cellular and molecular biology of SCs 8-10.

Peripheral nerves are unique in their tremendous elasticity. In physiological conditions, both peripheral nerve fibers and SCs are constantly subjected to sustained mechanical stresses from stretches and compression forces during the movement of the limbs 11, 12. In recent studies, the influence of mechanical stimuli on SC biology has been increasingly recognized 13, and there is a growing body of evidence that SCs are sensitive to the stiffness of their environments 14-16. It has been reported that mechanical stimuli can influence the cellular and molecular biology of SCs depending on the different types of stimuli 13. Mechanical stimuli are capable of activating focal adhesion complexes, and regulating important signaling pathways in SCs 17, 18. Similarly, shear stress on primary SCs has been shown to reduce the expression of myelin protein genes and promote the proliferation of SCs 14, 16. So far, many mechanotransduction pathways have been identified in SCs 13, and attempts have been made to promote nerve regeneration by mechanically stimulating SCs 19. Nevertheless, it is far from clear how SCs sense and respond to mechanical signals and consequent effect on nerve regeneration. Most previous studies primarily focused on the direct effect of mechanical stimuli on SCs. It is unknown whether mechanical stimuli can interfere with the intercellular communication between neurons and SCs providing essential support and modulating the homeostasis and plasticity in the neurogenerative process.

The functional and structural integrity of the nervous system is critically dependent on proper intercellular communication between neurons and SCs in the PNS 20. Several intercellular communications have been extensively studied in the nervous system, including ephaptic communication, paracrine signaling, and physical coupling 21, 22. In recent studies, extracellular vesicle (EV)-mediated communication between SCs and neurons has gained momentum 21. EVs, including microvesicles, ectosomes, exosomes, apoptotic bodies, *etc.*, are highly heterogeneous membranous vesicles with a lipid bilayer carrying various cargoes 23. It has been demonstrated that SC-derived EVs contain mRNA, miRNA, and protein cargoes, whose transfer into damaged axons is crucial for axonal elongation and remyelination 24. In particular, it has been shown that EV-derived miRNAs from SCs are capable of promoting axonal regeneration 25, indicating the critical role of SC-derived EVs in the intercellular communication between neurons and SCs in the context of peripheral nerve regeneration.

Mechanical stimuli are capable of modulating intercellular communication through EVs between various cells 26; one example is of communication between mesenchymal stem cells (MSCs) and human dermal microvascular endothelial cells to promote angiogenesis via up-regulation of matrix metalloprotease 2, transforming growth factor beta1, and basic fibroblast growth factor in secretomes from MSCs 27. Mechanical stress on osteocytes is capable of regulating bone metabolism by directly controlling the release of osteocyte-specific protein through EVs 28. These examples indicate that EVs constitute a response to mechanical stimuli in various cells. However, for SCs, which are sensitive to mechanical stimuli, it is unknown how SC-derived EVs sense and transduce mechanical signals. The role of EVs derived from mechanically stimulated SCs in axonal elongation and regeneration and the underlying mechanisms also remain unclear. Answers to these questions might provide new insights into understanding the mechanobiology of SCs during axonal regeneration and open new avenues for exploring a therapeutic approach to promote nerve regeneration after nerve injury.

In this study, we first established an effective magnetic force-induced-mechanical stimulation (MS) system of SCs (MS-SCs) and subsequently purified EVs from the conditioned culture supernatant. The effect of EVs derived from MS-SCs (MS-SCs-EVs) on axonal growth and nerve regeneration was examined *in vitro* and *in vivo*, and compared to that of EVs derived from non-stimulated SCs (SCs-EVs). Furthermore, we used next-generation sequencing to identify the differential miRNA profiles of MS-SCs-EVs and SCs-EVs. Our analysis identified specific miRNAs, especially miR-23b-3p, that were significantly altered in the MS-SCs-EVs. We investigated the role of MS-SC-EV-derived miR-23b-3p and demonstrated that miR-23b-3p enhanced neurite outgrowth *in vitro* and nerve regeneration *in vivo* by targeting neuropilin 1 (Nrp1) in neurons.

## Methods and Materials

### Isolation and characterization of SCs

SC primary cultures of sciatic nerves and brachial plexus were harvested from postnatal day 1-2 (P1-2) newborn Sprague-Dawley (SD) rats (provided by the Experimental Animal Center of the Fourth Military Medical University) following our established protocols 29. All experimental procedures were conducted under a protocol in accordance with the Guide for the Care and Use of Laboratory Animals (National Institutes of Health Publication No 85-23, revised1985) and approved by the Animal Research Committee of The Fourth Military Medical University, People's Republic of China. The primary SC cultures were stained by double immunofluorescence using NGF receptor p75 (p75^NTR^, ab52987; Abcam Inc., UK) and S100 protein (ab52642; Abcam) antibodies. The cell nuclei were stained with 4',6-diamidino-2-phenylindole (DAPI) solution (Sigma-Aldrich). The purity of primary SC cultures was determined by counting the number of p75^NTR^ and S100 double-positive cells and DAPI-labeled cells (Figure S1). The final preparations consisted of highly purified (>96%) SCs. The primary SC cultures were passaged no more than 3 times.

### Mechanical stimulation of SC cultures

The superparamagnetic iron oxide nanoparticles (SPIONs) (1 μg/μL) used in our study were purchased from Chemicell (Berlin, Germany) and were fabricated using Fe_3_O_4_ nanoparticles with a cationic polymer, branched polyethylenimine (PEI) (25 kDa). Surface-modified SPIONs were analyzed by transmission electron microscopy (TEM; H-600; Hitachi, Japan) and zeta potential/nanometer particle size analyzer (DelsaNano,Beckman Coulter, USA). The related magnetization information was obtained from Chemicell.

Primary SCs were cultured in T75 flasks in Dulbecco's Modified Eagle Medium (DMEM; Gibco, USA) containing 15% fetal bovine serum (FBS; Gibco, USA) until they reached 80-90% confluency. Prior to supplementing with SPIONs, SCs were rinsed with DMEM without serum, and the medium was replaced with fresh serum-free medium mixed with SPIONs and incubated at 37 °C under humidified 5% CO_2_. Subsequently, the cytotoxicity of SPIONs was evaluated by the Cell Counting Kit (CCK-8; Dojindo, Japan) according to the manufacturer's protocol. Briefly, different concentrations of SPIONs (0, 0.5, 1, 2, 4, 8 μg/mL) were added to SCs in a 96-well plate and incubated for 24 and 72 h. SCs were rinsed three times with PBS, then 100 μL fresh medium with 10 μL CCK-8 reagent was added to each well and incubated at 37 °C under humidified 5% CO_2_ for 4 h. The absorbance was measured at 450 nm by a microplate reader (Synergy H1, BioTek, USA).

After determining the optimal concentration of SPIONs by CCK-8 assays, SC cultures were then subjected to different intensities of the magnetic field (MF) to generate mechanical stimulation on SC cultures. The MS system consisted of an arc-shaped magnet twined with enamel-coated copper wire (diameter: 1.0 mm) and an MF generator with a proper frequency of 0-100 Hz and an intensity of 0-20 mT. The MF generator (GHY-III, patent ZL02224739.4; Fourth Military Medical University, Xi'an, China) was connected with the magnet to produce an open-circuit wave shape of MF. A Gaussmeter (Model 455 DSP; Lake Shore Cryotronics) was used to measure the accuracy of the MF output. A 2 Ω resistor was properly placed in series connection, and then the output frequency and waveform could be visualized on an oscillograph (6000 series; Agilent Technologies, Santa Clara, CA, USA). 50 Hz was demonstrated to be applicable for cell cultures, so the frequency of MF was set to 50 Hz in the subsequent experiments 30, 31. Flow cytometry was performed to evaluate the effect of different intensities of MF on cell apoptosis. SC cultures were co-incubated with SPIONs for 24 h, and then different MF gradients (0, 1, 2, 5, 10 mT) were applied to SC cultures for 24 h. The normal SC cultures without SPIONs were also subjected to different MF gradients (0, 1, 2, 5, 10 mT) as control. After MF exposure, SC cultures were maintained for another 24 h, and then the apoptosis rate of SCs was analyzed by flow cytometry. All samples were analyzed at 4 °C within 30 min.

### Electron microscopy analysis and quantification of intercellular SPIONs

To analysis the presence and localization of SPIONs, SCs treated with SPIONs were subjected to scanning electron microscopy (SEM) and transmission electron microscopy (TEM). Twenty-four hours after incubation with SPIONs, SCs were fixed and dehydrated with serial ethanol solutions. Then the samples were sputter-coated with gold and observed under a scanning electron microscopy (S-3400N, Hitachi, Japan). For TEM analysis, 24 h after SCs incubated with SPIONs, and then fixed and osmicated. The specimens were cut in 70 nm thin slices and observed under a transmission electron microscopy (TECNAI Spirit, FEI, USA).

SCs were co-incubated with SPIONs and the intercellular SPIONs were quantified by measuring the amount of iron in cells according to the method used previously 32. In brief, different concentrations of SPIONs (0, 0.5, 1, 2, 4, 8 μg/mL) were added to SCs and incubated for 48 h. Subsequently, the cultures were washed three times with sterile cold PBS supplemented with an iron chelator, deferoxamine (1 mM), to remove the extracellular SPIONs. The cells were detached, counted, and lysed in 27.75% HCl to dissolve the cellular components, and cellular lysates were diluted with deionized water (1:4) and filtered. The iron concentration was then measured by an inductive coupled plasma-atomic emission spectrometer (ICP-AES, ICPS-7500, Shimadzu, Japan).

### Isolation and purification of EVs

EVs were isolated and purified from the primary SC and MS-SC cultures following previously established protocol 33, 34. EVs were harvested from passage 2-3 primary SCs. In brief, 1.5×10^6^ SCs (or MS-SCs) were plated in a T75 flask and were cultured in DMEM/Nutrient Mixture F-12 (Gibico, USA) containing 10% FBS (Gibco, USA). After 24 h, the medium was replaced with 10 mL fresh DMEM/F-12 medium containing 10% exosome-free FBS (Xiaopeng, shanghai, China). Cultures were maintained for 48 h, and then the medium was collected and subjected to serial centrifugations. All procedures were conducted at 4 °C.

The EVs were characterized by TEM (TECNAI Spirit, FEI, USA) and Western blotting analysis. Nanoparticle tracking analysis (NTA) (ZetaView, Particle Metrix, Germany) was also performed to measure the concentration and size distribution of EVs. The NTA and BCA analysis (Beyotime Biotechnology, Shanghai, China) were performed to quantify the obtained EVs. In the Western blotting analysis to identify the EV markers, 2 μg of MS-SCs lysate and 2 μg of MS-SCs-EVs were loaded in each lane.

### Preparation of DRG explant and neuron cultures

DRGs were harvested and purified from neonatal (postnatal day 0-1) SD rats (provided by the Experimental Animal Center of the Fourth Military Medical University), as previously described 35. All experimental procedures were conducted under a protocol in accordance with the Guide for the Care and Use of Laboratory Animals (National Institutes of Health Publication No 85-23, revised1985) and approved by the Animal Research Committee of The Fourth Military Medical University, People's Republic of China.

DRG explants and neurons were seeded on coverslips pre-coated with rat tail collagen (Invitrogen, Carlsbad, CA) and maintained in the neurobasal medium (Gibco) supplemented with 50 ng/mL human nerve growth factor (hNGF) (R&D Systems, Minneapolis, MN), 2 mM L-glutamine (Gibco), 2% B27 (Gibco) and 1% penicillin-streptomycin.

In all experiments, DRGs were treated with 10 μM cytosine arabinoside (Sigma-Aldrich) to eliminate contaminated fibroblasts. To evaluate the effect of SCs-EVs and MS-SCs-EVs on axonal regeneration, 1×10^8^ SCs-EVs, MS-SCs-EVs or MS-SCs-EVs pretreated with distilled water, and an equal volume of PBS was added the next day after DRG explants and neurons were plated and cultured for 3 days. It has been reported that small iron oxide nanoparticles can be encapsulated by EVs or exosomes 36. To investigate the effect of SPIONs on SCs-EVs, DRG cultures were treated with EVs derived from SPIONs-treated SCs (SPIONs-SCs-EVs) to examine their effects on axonal growth. Immunocytochemistry using NeuN (1:500, Abcam) and β-tubulin Ⅲ (1:200, Abcam) antibodies was performed and the axonal length from the edge of DRG explants to the tip of axons, the area of the explant body and neurites, as well as the average length of 40 longest axons of DRG neurons were measured by Image-Pro Plus 6.0 (Media Cybernetics, Bethesda, MD, USA) software to evaluate axonal elongation as previously described 37.

### Labeling EVs with PKH26

Purified EVs obtained from MS-SC culture supernatants were labeled with the PKH26 kit (red) (Sigma-Aldrich) to evaluate the uptake of EVs as previously described 38. Briefly, 250 μL of purified MS-SCs-EVs were diluted in PBS and mixed with 250 μL of Diluent C. Next, 2 μL of PKH26 dye was added into 500 μL of Diluent C. The final PKH26 concentration was 1×10^-6^ M, which was mixed with the EV solution prepared above for 5 min at room temperature. One mL of 5% BSA was added to neutralize the excess dye, which was removed by centrifugation. The labeled EVs were re-suspended in sterile PBS and ultracentrifuged at 100,000 g for 70 min to pellet the PKH26-labeled EVs. The diluted PKH26 solution mixed with PBS was used as a negative control. PKH26 labeled MS-SCs-EVs were co-cultured with DRG neurons in serum-free medium overnight. The DRG neurons with internalized MS-SCs-EVs were immunostained with β-tubulin Ⅲ (1:200, Abcam) antibody or phalloidine-FITC (G-Clone, China) and for nuclear staining by DAPI (Sigma-Aldrich) and observed under a fluorescence confocal microscope (A1+, Nikon, Japan).

### Surgical procedures

The animal studies were conducted in accordance with the Guide for the Care and Use of Laboratory Animals (National Institutes of Health Publication No 80-23, revised 1996) and approved by the Animal Research Committee of The Fourth Military Medical University, People's Republic of China. Adult SD male rats weighing 200-240 g (purchased from the Laboratory Animal Center of the Fourth Military Medical University) were housed in a specific pathogen-free laboratory animal center with controlled temperature (24±1 °C) and 12:12 h light: dark cycle.

All animals were randomly divided into four groups: the autologous graft (autograft), conduit (control), SCs-EVs+conduit (SCs-EVs), and the MS-SCs-EVs+conduit (MS-SCs-EVs) groups. Animals were anesthetized intraperitoneally with pentobarbital sodium (1%, 40 mg/kg animal weight). The left sciatic nerve was exposed in a sterile environment, and a 5-mm gap in the nerve was created. In the autograft group, the 5-mm amputated nerve segment was reversed 180º and resutured under the microscope. In the other three groups, the nerve conduits were used to bridge the nerve defect and sutured to the proximal and distal ends of nerve stumps by three perineural 10/0 nylon sutures. The nerve conduit was filled with 3.6×10^9^ SCs-EVs, MS-SCs-EVs and an equal volume of PBS for the SCs-EVs, MS-SCs-EVs and control groups. Furthermore, to investigate the effects of miR-23b-3p on neurite outgrowth *in vivo*, we applied 3.6×10^9^ EVs derived from miR-23b-3p knocked-down MS-SCs (miR-23b-3p KD MS-SCs-EVs) with the PCL conduit to the sciatic nerve injury rats. The relationship of neuronal Nrp1 expression with MS-SCs-EVs derived miR-23b-3p was examined *in vivo*. In this experiment, 18 rats were randomly divided into three groups; all animals were subjected to the operations to create a sciatic nerve injury of the left legs. In the MS-SCs-EVs and miR-23b-3p KD MS-SCs-EVs groups, 3.6×10^9^ MS-SCs-EVs and miR-23b-3p KD MS-SCs-EVs were injected into the injury site, respectively. In the control group, the equal volume of PBS was injected in the injury site.

Poly (ε-caprolactone) (PCL) has gained increasing attention in regenerative medicine because of its suitable mechanical strength, toughness, biodegradability, and cytocompatibility. In addition, the potential use of PCL conduit has been proposed in supporting nerve regeneration 39. In this study, we used PCL conduit to bridge the nerve gap, which was mainly to take advantage of its mechanical strength and toughness. Nerve conduit was prepared following the procedures described previously 40. Briefly, 15% (w/v) PCL solution was prepared by dissolving the PCL pellets (Mn = 80 000) (Sigma-Aldrich) in a mixture of methanol and chloroform (1:5 v/v) (Sigma-Aldrich). PCL solution was delivered at a flow rate of 4.5 mL/h by a syringe pump (Cole Parmer, USA) in a 10 mL syringe with 21-gauge blunt tip syringe needle. Twelve kV voltages were applied to the electro-spun system with 18 cm distance between collector and syringe tip. A 10 cm long and 1.5 cm in diameter rotating stainless steel rod was designed as a collector to receive and fabricate the PCL nerve conduit; the newly fabricated conduit was dried in vacuum to eliminate the residual PCL solvent at room temperature for 24 h.

### Immunofluorescent staining

Immunofluorescence was performed according to the previously described protocols 41. The cultures were rinsed and fixed with 4% paraformaldehyde for 15 min, permeabilized with 0.2% Triton X-100 for another 15 min at room temperature, and blocked for 1 h in 10% normal goat serum at room temperature. Cultures were co-incubated with primary antibodies overnight at 4 °C and reacted with fluorescent conjugated secondary antibodies for 1 h at 37 °C. All procedures were performed by rinsing in PBS for 5 min and repeated three times. The DRG explant was stained with β-tubulin III (1:200, Abcam) antibody and DRG neurons were stained for double immunofluorescence using β-tubulin III (1:200, Abcam) and NeuN (1:500, Abcam) antibodies.

Four weeks after the operation, longitudinal and cross sections of the regenerated nerve segments were cut using a paraffin section system. The sections were stained with mouse anti-NF 160 antibody (1:200, Abcam) and reacted with goat anti-mouse IgG FITC (1:200; Abcam) secondary antibodies. All procedures were performed by rinsing three times in PBS for 5 min each.

The left DRGs (L4-6) were harvested seven days after surgery, fixed in 4% paraformaldehyde for 4 h, and dehydrated in 30% sucrose for 24 h at 4 °C. Finally, the DRGs were sectioned using a paraffin section system. The sections were stained with mouse anti-β-tubulin III (1:200, Abcam) and rabbit anti-neuropilin 1 (1:250, Abcam) antibodies followed by incubation with goat anti-mouse IgG FITC (1:200; Abcam) and goat anti-rabbit IgG cy3 (1:200; Abcam) secondary antibodies, respectively. All procedures were accompanied by rinsing three times in PBS for 5 min each.

### miRNA sequencing

EVs were isolated from SC and MS-SC cultures, as previously described 33, 34. Total RNA extracted from EVs was used for miRNA sequencing. miRNA sequencing and library preparation were performed by Ribobio Co. Ltd (Guangzhou, China). In brief, total RNA was extracted from EVs and fractionated, and only small RNAs ranging from 18 to 30 nts were selected for library construction. After amplification by PCR, products were sequenced using the Illumina HiSeq 2500 platform. Raw reads were collected using the Illumina analysis software.

### Cell transfection, lentiviral, RNA oligoribonucleotides and plasmid packaging

Synthetic miRNA mimics, mimic control duplexes, miRNA inhibitors, inhibitor-NC, siRNA for Nrp1 and siRNA nonspecific control duplex were synthesized and purified by Sangon Biotech (Shanghai, China). Plasmid lacking the 3'-UTR of Nrp1 and the Nrp1 NC plasmid were designed by Genechem (Shanghai, China). The siRNA sequences are shown in Table S1. RNA oligonucleotides were transfected by using Lipofectamine 3000 (Invitrogen, California, USA) following the manufacturer's instructions. Antisense oligonucleotides (ASO) of miR-23b-3p lentivirus gene transfer vector were constructed by Genechem (Shanghai, China) to knock down miR-23b-3p expression in SCs. The sequence of the ASO was 5'-GGTAATCCCTGGCAATGTGAT-3', which was against the mature sequence of miR-23b-3p. The oligonucleotides were annealed and cloned into Agel and EcoRI sites of lentiviral vector GV280 42.

### Quantitative real-time PCR (qRT-PCR) and Western blotting

The expression of miR-26a-5p, miR-1291-3p, miR-186-5p, miR-23b-3p, and miR-365-3p in SCs-EVs and MS-SCs-EVs was evaluated by qRT-PCR, which was also used to compare the expression of the target gene Nrp1. EV miRNA was isolated by using the exoRNesay serum/plasma starter kit (QIAGEN, Hilden, German). miDETECT A Track^TM^ miRNA qRT-PCR primer sets (one RT primer and a pair of qPCR primers for each set) specific for miR-26a-5p, miR-1291-3p, miR-186-5p, miR-23b-3p, and miR-365-3p were designed by RiboBio (Guangzhou, China). Small nuclear RNA RNU6B (U6) was used as an internal control. For Nrp1 mRNA expression analysis, total RNA was extracted from cells using RNAiso Plus (Takara, Dalian, China) and qRT-PCR was performed using the miDETECT A Track miRNA qRT-PCR Kit (RiBoBio, Guangzhou, China) following the manufacturer's protocol. β-actin was used as an internal control. The relative standard curve method (2^-ΔΔ Ct^) was used to determine the relative expression. The primer sequences are shown in Table S2. All measurements were performed in triplicate.

The Western blot protocol was carried out as previously described 43. The following antibodies were used in our experiments: mouse anti-Alix (#2171; Cell Signaling Technology, Danvers, MA, USA), mouse anti-TSG101 (ab83; Abcam), mouse anti-CD63 (CBL553, Sigma-Aldrich), mouse anti-p75^NTR^ (ab52987; Abcam), rabbit anti-calnexin (ab10286, Abcam), and rabbit anti- Neuropilin 1 (ab81321, Abcam). Goat anti-rabbit and goat anti-mouse secondary antibodies (Cwbiotech, Beijing, China) were used at a 1:10000 dilution.

### Luciferase reporter assay

Luciferase reporter assays were performed following the manufacturer's protocol. 293 T cells were seeded in a 24-well plate and co-transfected with luciferase reporter constructs encoding the wild-type 3'-UTR region of Nrp1 (Nrp1-3'-UTR-WT) or a mutated Nrp1 3'-UTR region (Nrp1-3'-UTR-MUT), and miR-26a-5p, miR-129-1-3p, miR-186-5p, miR23b-3p, and miR-365-3p mimics (50nM), or Non-target Control (NC) (Ribobio, Guangzhou, China) using Lipo6000^TM^ (Beyotime). Luciferase activities were measured using a Dual-Luciferase Reporter Assay Kit (Promega, Madison, WI, USA) according to the manufacturer's protocol. Each experiment was performed in triplicate.

### Statistical analysis

The data are expressed as means± standard error of mean (SEM). Statistical tests were conducted using GraphPad Prism 8 software (GraphPad, USA). Statistical significance was assumed at values of *P*<0.05.

## Results

### MS of SCs cultures

The particle size of SPIONs was 21.7±2.3 nm, as determined by TEM (Figure S2A), and the saturation magnetization information of SPIONs at 3.382 Qe was 61.62 emu/g (Figure S2B). SPIONs showed an average diameter of 121.7±17.4 nm and a positive charge of 18.94±2.9 mV as measured by a ZetaPALS particle analyzer (Figure S2C-D). The discrepancy between the average diameter of SPIONs as determined by TEM and ZetaPALS can be explained by the agglomeration of charged SPIONs during the preparation of sample solution for ZetaPALS analysis. These results demonstrated that the SPIONs were positively charged and suitable for cellular internalization.

**Figure 1:**
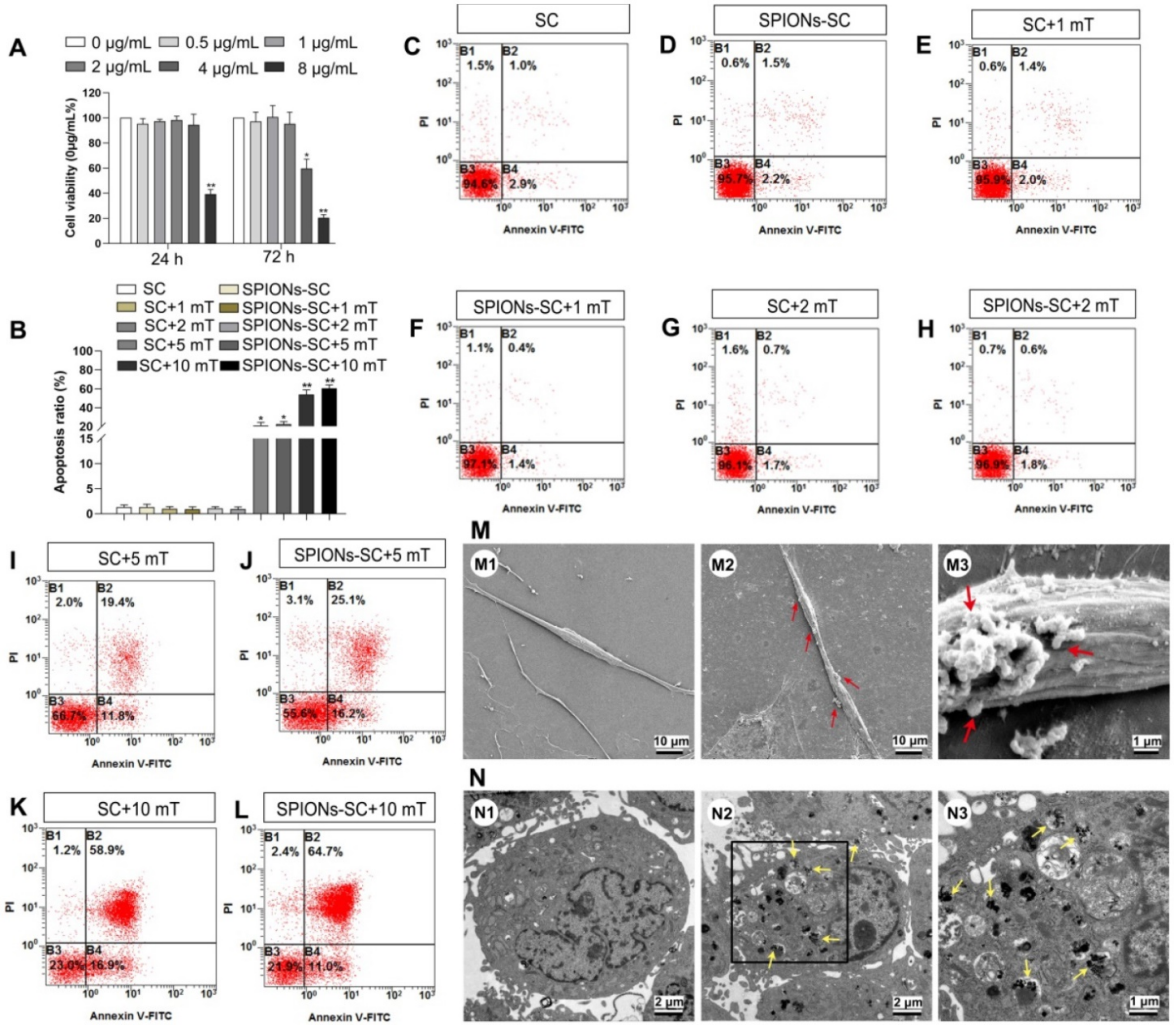
** Cytotoxicity and apoptosis of SCs and cellular localization of SPIONs.** (**A**) Effect of different concentrations of SPIONs on cell viability was examined by CCK-8 assay (n=3) (**B**) Percentage of apoptotic cells at different MF intensities was evaluated by flow cytometry (n=3) (**C**) SC cultures (**D**) SC cultures with 2 μg/mL SPIONs (**E**) SC cultures +MF group (1 mT) (**F**) SC cultures with 2 μg/mL SPIONs+MF group (1 mT) (**G**) SC cultures +MF group (2 mT) (**H**) SC cultures with 2 μg/mL SPIONs+MF group (2 mT) (**I**) SC cultures +MF group (5 mT) (**J**) SC cultures with 2 μg/mL SPIONs+MF group (5 mT) (**K**) SC cultures +MF group (10 mT) (**L**) SC cultures with 2 μg/mL SPIONs+MF group (10 mT) (**M**) Representative SEM images of normal control SCs (M1) and SCs incubated with 2 μg/mL SPIONs for 24 h (M2, M3). The red arrows point SPION agglomerates on the cell membrane. (**N**) Representative TEM images of normal control SCs (N1) and SCs incubated with 2 μg/mL SPIONs for 24 h (N2). The yellow arrows point SPIONs in the cytoplasm; most retained SPIONs dispersed as single particles. (N3) High magnification of the boxed area in (E). All data are expressed as means±SEM. One-way analysis of variance (ANOVA) test with Tukey's post hoc test was used to examine the significance of results. * P<0.05 and ** P<0.01 for comparison with SCs cultures control group.

After 24 h and 72 h of co-incubation with SPIONs, SC cultures were subjected to CCK-8 assay to evaluate the cytotoxicity of SPIONs in SCs. No significant change was observed in cell viability when SCs were subjected to SPIONs at concentrations of 0, 0.5, 1, 2 and 4 μg/mL for 24 h or at 0, 0.5, 1, and 2 μg/mL for 72h after co-incubation. However, cell viability significantly decreased by 36.9% at 4 μg/mL when the incubation time was prolonged to 72 h (Figure 1A). Therefore, SPIONs at 2 μg/mL was used as a safe concentration to avoid the potential cytotoxicity for SCs. Furthermore, the effect of MF on the survival of SCs or SCs loaded with 2 μg/mL SPIONs (SPIONs-SCs) was investigated after 48 h of MF exposure by flow cytometry (Figure 1B-L). The apoptosis ratio of SPIONs-SCs and SCs at 1 mT, 2 mT was similar to that without MF. However, the apoptosis rate of SPIONs-SCs and SCs was significantly increased to 22.53%±1.50 and 21.07%±1.82, respectively, when the MF exposure increased to 5 mT. When the MF increased to 10 mT, the apoptosis rate of SPIONs-SCs and SCs increased further to 60.70%±2.01 and 53.9%±2.83, respectively (Figure 1B). These results indicated that MF with an intensity higher than 5 mT is detrimental to SPIONs-SCs and SCs.

The mechanical tension applied to SCs could be regulated by adjusting the concentration of SPIONs added to SC cultures and the intensity of the external MF. More precisely, we could quantify intracellular SPIONs and calculate the magnetic forces acting upon SCs by physical methods. The intracellular SPIONs were quantified by measuring the amount of iron in the cells (Table 1). Twenty-four hours after the incubation of SCs with SPIONs (2 μg/mL), SEM analysis revealed that some particles had been attached on the cell surface, which was not detected in the control SCs (Figure 1M). In addition, TEM analysis showed that SPIONs had been internalized by SCs and localized in the cytoplasm of SCs (Figure 1N). The presence of intracellular SPIONs was also confirmed by quantifying intercellular iron, which increased proportionately with SPION concentrations (Table 1). The total force acting upon a single SC can be calculated by physical methods, which have been previously described 35, 44. If the SPION concentration was too low, the force upon SCs was negligible. Therefore, SPIONs at 2 μg/mL, namely the intracellular iron 1.27±0.08 pg/per cell, and MF at 2 mT were applied to mechanically stimulate SCs in the subsequent experiments.

### Characterization and internalization of EVs derived from MS- SCs

**Figure 2:**
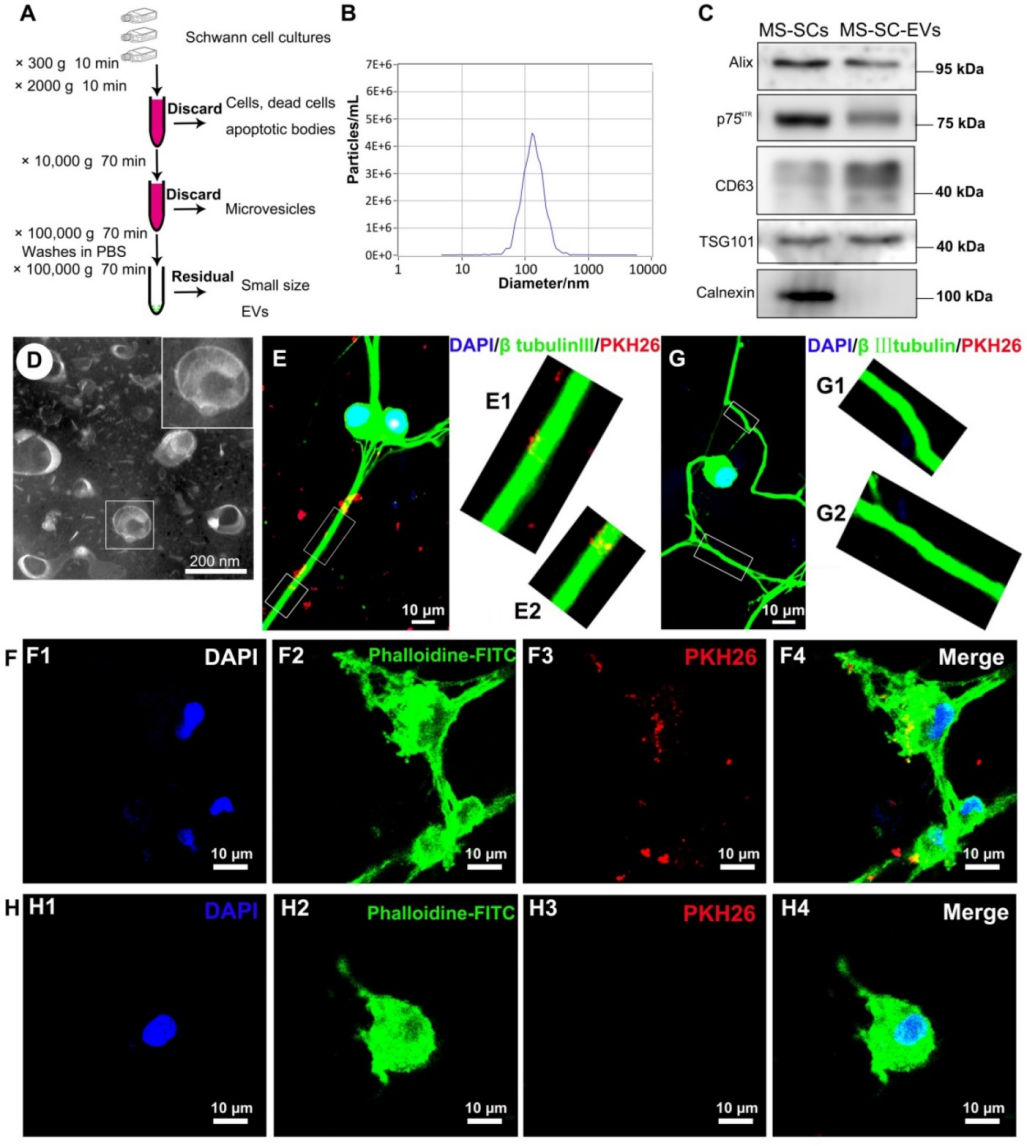
** Characterizations of MS-SCs-EVs and their internalization by DRG neurons.** (**A**) Isolation and purification procedures of EVs derived from SC supernatants (**B**) Representative traces from NTA (**C**) Western blot analysis of MS-SCs-EVs confirmed the presence of EV marker proteins of Alix, CD63, TSG101, and the absence of non-exosomal protein (calnexin), the marker protein of SC, p75^NTR^ , was also detected in the EVs derived from MS-SC cultures, equal amount (2 μg) of protein was loaded in each lane, and MS-SCs lysate was used as a control for surface markers (**D**) TEM analysis of isolated EVs. The MS-SCs-EVs were labeled with PKH26 (red), and DRG neurons were immunofluorescent stained for β-tubulin III (green) or cytoskeletal staining by phalloidine (green) with DAPI nuclear counterstaining (blue). Representative images showed the presence of labeled EVs (red) within the axons and cytoplasm of DRG neurons in the MS-SCs-EV-treated groups (**E and F**), while no EVs were observed in the axons and cytoplasm of DRG neurons (**G and H**) in the control groups. Scale bars: (D) 200 nm (E-H) 10 μm.

Both SCs and SPIONs-SCs were subjected to MF (2 mT) for 24 h. The EVs were isolated from SC and MS-SC culture supernatants and purified following standard protocols (Figure 2A). The MS-SCs derived EVs (MS-SCs-EVs) showed a modal peak of 137.52±1.77 nm (Figure 2B), which was directly traced by NTA. Studies have shown that ultracentrifugation can cause aggregation of EVs 45, 46, and the aggregated EVs labeled by PKH26 showed a quite large diameter, which seemed inconsistent with the NTA results. The number of the obtained particles measured by NTA was (7.10±0.404)×10^10^/flask from SCs and (7.27±0.504)×10^10^/flask from MS-SCs. Further studies showed that the exosomal markers Alix, CD63, and TSG101 were expressed in MS-SCs-EVs, while calnexin, an integral protein was not detected in MS-SCs-EVs (Figure 2C). Interestingly, we found that MS-SCs-EVs expressed p75^NTR^, which is a biomarker of dedifferentiated SCs (Figure 2C) and shows diverse functions in the PNS. The detection of p75^NTR^ in MS-SCs-EVs further confirmed the origin of the EVs and indicated the regulatory role of MS-SCs-EVs in PNS. Furthermore, the cup-shaped morphology of the preparations was observed by TEM, indicating that these vesicles displayed characteristic features of EVs (Figure 2D).

Upon release, EVs interact with recipient cells by surface contact and are internalized by endocytosis or fusion with the plasma membrane of recipient cells 47. In this study, to investigate whether MS-SCs-EVs could be internalized by DRG neurons, MS-SCs-EVs were labeled with PKH26 and incubated with DRG neurons. After incubation for 8 h, the PKH26-labeled MS-SCs-EVs were detected in the distal axons (Figure 2E, E1 and E2) and cell bodies (Figure 2F, F1-4) of DRG neurons, in contrast to the absence of staining in the negative control group (Figure 2G, G1, G2, H, H1-4). These results indicated that the MS-SCs-EVs can be internalized by DRG neurons.

### MS-SCs derived EVs enhance axonal growth *in vitro*

**Figure 3:**
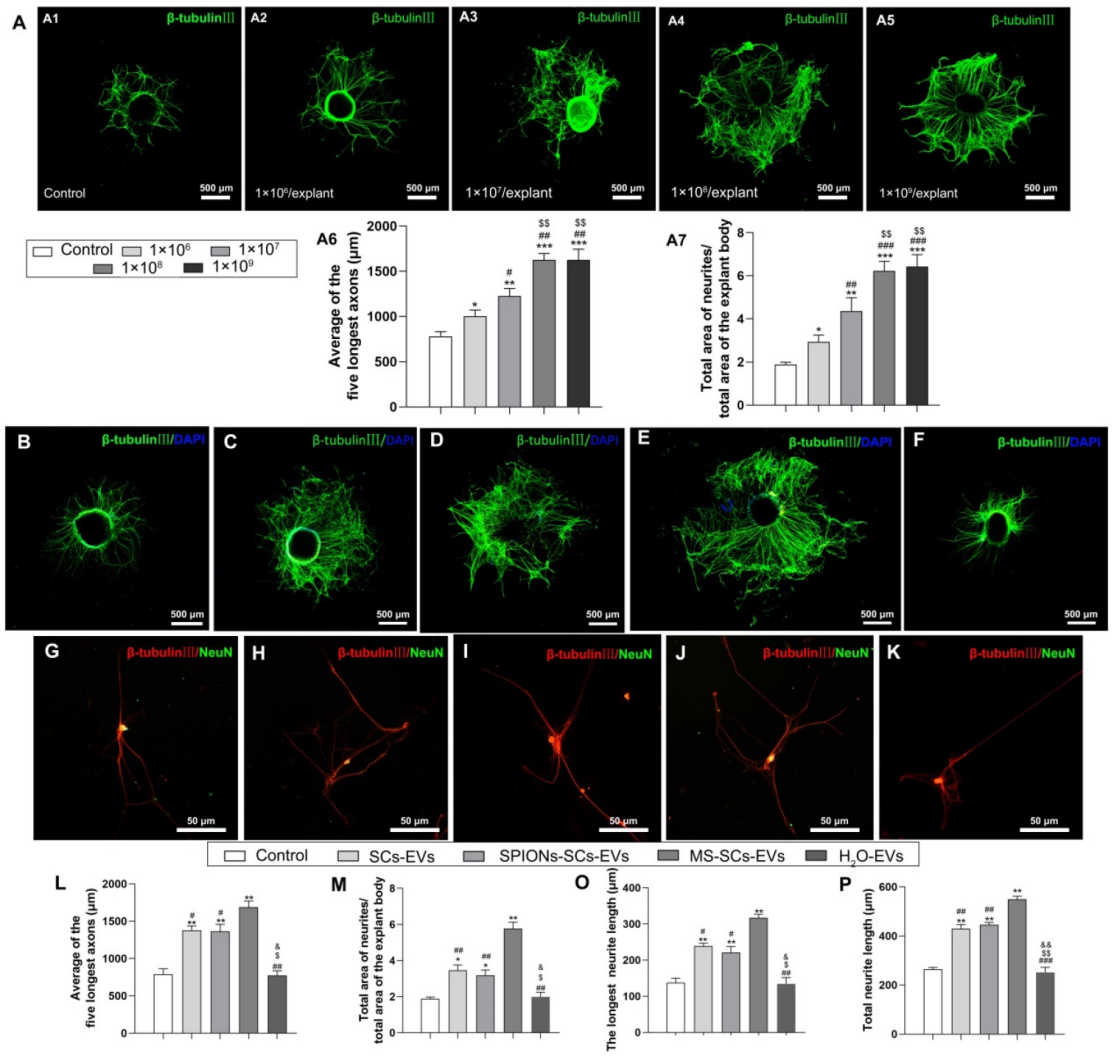
** MS-SCs-EVs enhance axonal growth of DRG explants and neurons.** Representative images of DRG explants stained for β-tubulin III (green) with nuclear DAPI counterstaining, DRG neurons were stained for β-tubulin III (red) and NeuN (green). (**A**) Representative images of DRG explants after the addition of PBS plus increasing MS-SCs-EVs concentrations (A1-A5). (A6 and A7) Axonal lengths were quantified and plotted for each MS-SCs-EVs concentration group of three independent experiments (n =4 per group). Axonal elongation of DRGs after 3 days in PBS (**B and G**), SCs-EVs (**C and H**), SPIONs-SCs-EVs (**D and I**), MS-SCs-EVs (**E and J**), MS-SCs-EVs pre-treated with H_2_O (**F and K**). (L-K) Axonal lengths were quantified and plotted for each treatment of three independent experiments (L-M n=5 per group; O-P n=20 per group). Scale bars: (A1-A5, B-F) 500 μm; (G-K) 50 μm. All data are expressed as means±SEM. One-way ANOVA test with Tukey's post hoc test was used to examine the significance of results. (**L-P**) * P<0.05 and ** P<0.01 for comparison with the PBS control group; #* P*<0.05 and ##* P*<0.01 for comparison with the MS-SCs-EVs group; $* P*<0.05 and $$* P*<0.01 for comparison with the SCs-EVs group; & *P*<0.05 and && *P*<0.01 for comparison with the SPIONs-SCs-EVs group. (A6 and A7) * P<0.05, ** P<0.01 and *** P<0.001 for comparison with the MS-SCs-EVs (0 μg/mL) group; #* P*<0.05, ##* P*<0.01 and ###* P*<0.001 for comparison with the MS-SCs-EVs (1 μg/mL) group; $* P*<0.05, $$* P*<0.01 and $$$* P*<0.001for comparison with the MS-SCs-EVs (2 μg/mL) group.

SCs-EVs have been reported to enhance axonal elongation 24. To investigate the effect of MS-SCs-EVs on neurite outgrowth* in vitro*, we examined their effect on DRG explant and purified neurons. First, we analyzed the effect of different concentrations of MS-SCs-EVs on neurite outgrowth in DRG explants. As shown in Figure 3A, the beneficial effect of MS-SCs-EVs on neurite outgrowth in DRG explant was dose-dependent, reaching a plateau at 1×10^8^ MS-SCs-EVs per explant (Figure 3A1-A7). Furthermore, compared with the control group, the axon length increased significantly when SC-EVs (1×10^8^ per explant) were applied to the DRG explant together with SPION-SC-EVs (1×10^8^ per explant) (Figure 3B-D), which was consistent with previous findings 24. Importantly, the MS-SCs-EVs (1×10^8^ per explant) could further promote neurite outgrowth in DRG explants (Figure 3C, E). The average length of five longest axons in MS-SC-EV group (1687.17±40.71 μm) was significantly higher than that in the SC-EVs group (1377.75±30.19 μm) and control group (788.54±37.04 μm), however, the enhanced neurite outgrowth by MS-SCs-EVs was abolished by pre-treatment with H_2_O, indicating that the effect was dependent on the EV integrity (Figure 3B-F). As expected, the enhancement was abolished when MS-SCs-EVs were pre-treated with H_2_O (774.51±30.96 μm) (Figure 3L). Also, no significant difference was found between SCs-EVs and SPIONs-SCs-EVs on enhancing neurite growth (Figure 3L).

It has been speculated that large-sized DRG explants contain more neurons, which tend to have more neurites. To minimize the potential interference from the size of DRG explants, the ratio of the total area of neurites to the total area of the explant body was quantified. In the MS-SCs-EVs group, the ratio of neurites area to explant body area was 5.77±0.18, which was significantly larger than that in the SCs-EVs, SPIONs-SCs-EVs and control groups (3.46±0.15, 3.18±0.14 and 1.89±0.04, respectively) (Figure 3M).

Next we examined the effect of MS-SCs-EVs (1×10^8^ per well) by evaluating the neurite length of purified DRG neurons (Figure 3G-K). The longest neurite length in the MS-SCs-EVs group (317.25±4.80 μm) was significantly higher than that in the SCs-EVs (239.00±3.92 μm), SPIONs-SCs-EVs (221.25±8.25 μm) and PBS control (137.75±6.25 μm) groups. Again, the enhancement of MS-SCs-EVs on neurite outgrowth in purified DRG neurons was abolished by pre-treatment with H_2_O (Figure 3O). Also, the total neurite length was highest in the MS-SCs-EVs group, followed by SCs-EVs and SPIONs-SCs-EVs groups, and lowest in the PBS control and EVs-H_2_O groups; SPIONs had no significant effect on SCs-EVs' enhancement of neurite outgrowth (Figure 3P). These results demonstrated that MS-SCs-EVs are capable of further promoting neurite outgrowth *in vitro* when compared to SCs-EVs, and the enhancement on neurite outgrowth is dependent on the integrity of EVs.

### MS-SCs -EVs promote nerve regeneration in vivo

**Figure 4:**
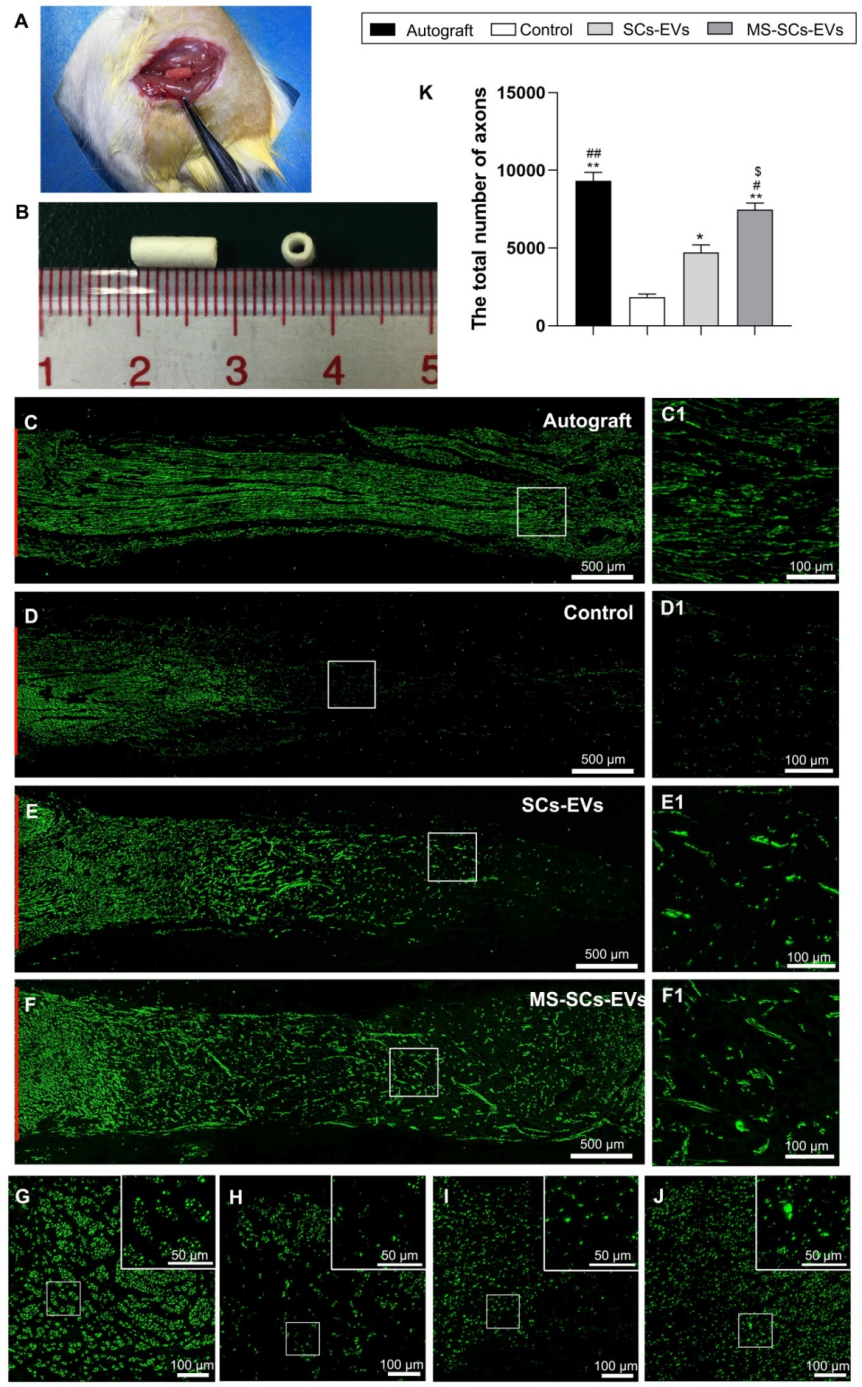
** MS-SCs-EVs promote axonal regeneration following sciatic nerve injury.** (**A**) Sciatic nerve graft conduit after implantation (**B**) Gross appearance of the graft conduit. Four weeks after surgery, representative images of regenerated axons stained for NF 160 (green) in the autograft group (**C**), PBS control group (**D**), SCs-EVs group (**E**), MS-SCs-EVs group (**F**); (C1-F1) is the higher magnification of the boxed area of (C-F). Cross-section of the distal regenerated nerve segments at 4 weeks after surgery. Representative images of regenerated axons in the distal nerve segment stained for NF 160 (green) in the autograft group (**G**), PBS control group (**H**), SCs-EVs group (**I**), MS-SCs-EVs group (**J**); the upper right corner of the images (G-J) is higher magnifications of the boxed area. Quantification of the total number of the regenerated axons in the cross-sections at 4 weeks after injury (K); n = 4 per group, scale bars: (C-F) 500 μm, (G-J) 100 μm. Results are expressed as the mean ± SEM. One-way ANOVA test with Tukey's post hoc test was used to examine the significance of results. * P<0.05 and ** P<0.01 for comparison with the PBS control group, #* P*<0.05 and ##P<0.01 for comparison with the SCs-EVs group; $* P*<0.05 for comparison with the autograft group.

After demonstrating that MS-SCs-EVs promoted neurite outgrowth *in vitro*, we evaluated their effect on axonal regeneration in a rat model of peripheral nerve injury. A 5-mm sciatic nerve defect was generated in rats and bridged with an autograft or a PCL nerve conduit containing an equal volume of PBS, SCs-EVs or MS-SCs-EVs (Figure 4A and B). Four weeks following injury, robust axonal regeneration was observed in the MS-SCs-EVs and autograft groups when compared to the SCs-EVs and PBS groups, demonstrating that MS-SCs-EVs were capable of promoting axonal regeneration after sciatic nerve injury *in vivo* (Figure 4C, D, E and F). The regenerated axons were evenly distributed from the proximal to the distal end of the graft segment in the MS-SCs-EVs and autograft groups (Figure 4C1, F1). In contrast, regenerated axons were rarely found in the distal end of the graft in the SCs-EVs and PBS groups (Figure 4D1, E1). The regenerated axons in the cross-section of the distal stump were quantified at 4 weeks after injury (Figure 4G-J). The number of NF-160-positive axons in autograft (9328.25±267.66) and MS-SCs-EVs groups (7466.25±216.69) were significantly higher than that in the PBS (1848.75±95.68) and the SCs-EVs groups (4720.00±243.02), indicating that axons successfully regenerated in the distal stump in the autograft and MS-SCs-EVs groups (Figure 4K).

### The miRNA expression profiles in MS-SCs-EVs

**Figure 5:**
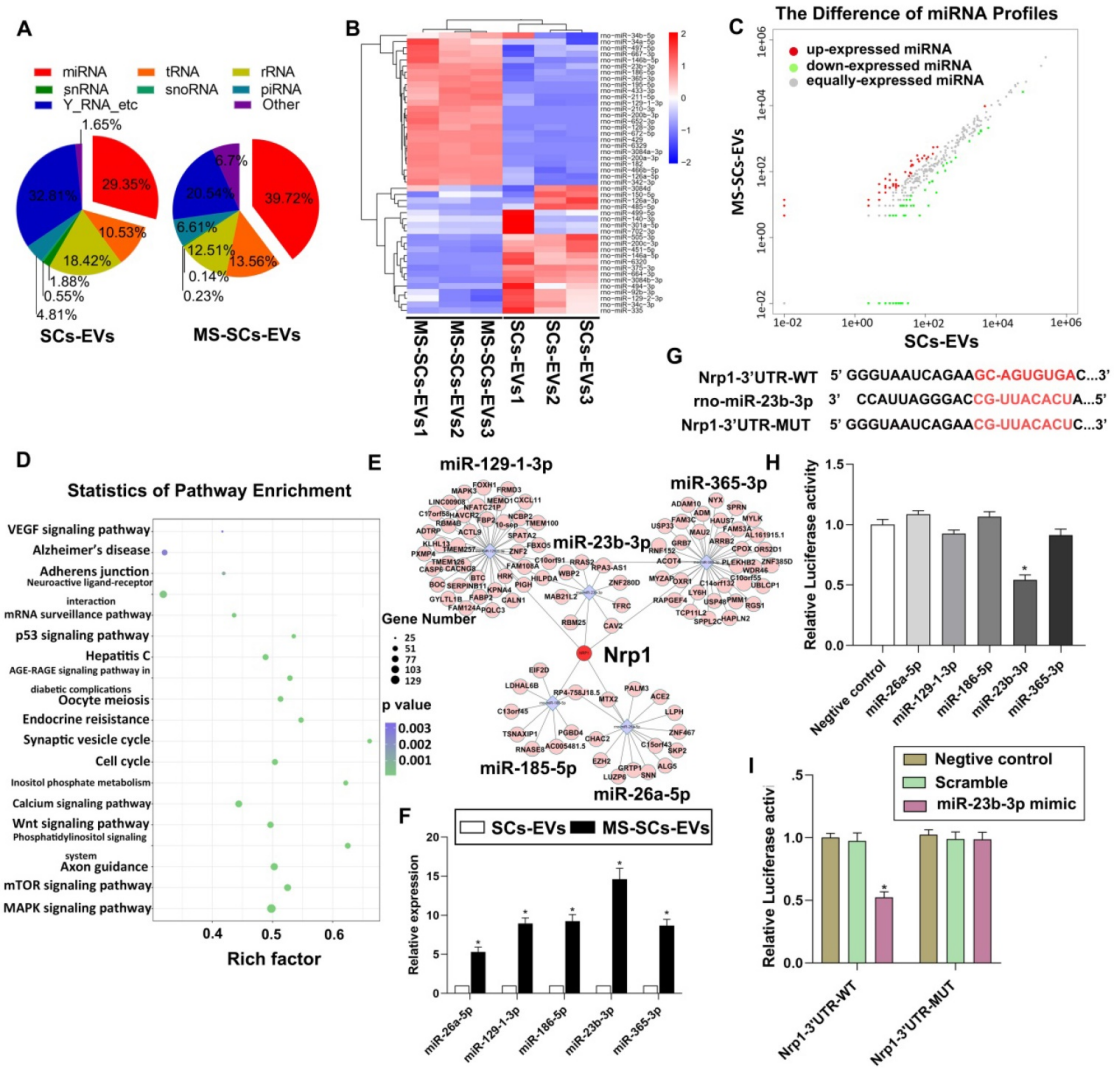
** miRNA expression profile of MS-SCs-EVs is significantly altered compared with SCs-EVs.** (**A**) Percentage of small RNA categories in all reads mapped to noncoding RNA databases. (**B**) Heatmap diagram of differential miRNA expression between MS-SCs-EVs and SCs-EVs. Gene expression data were obtained using next-generation sequencing on the Illumina HiSeq 2500 platform. Expression values shown are mean centered. Red, increased expression; blue, decreased expression, and white, mean value. (**C**) Differentially expressed miRNAs between MS-SCs-EVs and SCs-EVs. Red, increased expression; green, decreased expression; gray, no difference. P < 0.05 and fold change>2 were considered significant. (**D**) KEGG pathway enrichment of target genes of the predicted differentially expressed miRNAs. Advanced bubble chart shows enrichment of differentially expressed genes. Y-axis represents pathways, and the x-axis represents rich factor, depicting the ratio of the differentially expressed genes enriched in the pathway and the amount of all genes annotated in this pathway. Size and color of the bubble represent the amount of differentially expressed genes enriched in the pathway and enrichment significance, respectively. (**E**) Competing RNA network with the most significantly altered miRNAs. (**F**) Confirmation of the differentially expressed miRNAs in MS-SCs-EVs and SCs-EVs by qRT-PCR (n=6). (**G**) Predicted miR-23b-3p binding site in the 3'-UTR region of Nrp1. A mutated miR-23b-3p binding site was generated in the complementary site for the seed region of miR-23b-3p. (**H**) Luciferase assay was performed 48 h after transfection using the dual luciferase. MiR-23b-3p down-regulated the luciferase activity compared with the negative control and other miRNAs. (**I**) miR-23b-3p mimic down-regulated the luciferase activity controlled by wild-type Nrp1 3'-UTR, while luciferase activity controlled by mutant Nrp1 3'-UTR was not affected. Student's t-test and one-way ANOVA test with Tukey's post hoc test, * P<0.05 for comparison with the SCs-EVs group or the negative control group.

The EV-enriched miRNA content was recognized to be critical in intercellular communication in the nervous system 25. To explore the role of miRNAs in the beneficial effect of MS-SCs-EVs on axonal elongation, we next identified the miRNA expression profiles of SCs-EVs and MS-SCs-EVs using Illumina HiSeq 2500 high-throughput sequencing (miRNA-seq). After trimming low-quality reads, contaminants, adaptors, and reads smaller than 17 nt, the remaining reads were mapped to noncoding (nc) RNA databases. The percentage of miRNAs in the total RNA extracted from SCs-EVs and MS-SCs-EVs corresponded to 29.35% and 39.72%, respectively (Figure 5A). We next compared the expression level of miRNAs in SCs-EVs and MS-SCs-EVs (Figure 5B). Using a fold change of >2 and *P*<0.05 as the threshold cutoff, we f detected 20 miRNAs (Table 2) that were significantly altered between SCs-EVs and MS-SCs-EVs (Figure 5C). A KEGG pathway enrichment analysis was performed to investigate the potential biological consequences of the altered miRNAs. Interestingly, we found that enrichment of pathways was related to axon growth and regeneration, including axon guidance, Wnt signaling, ErbB pathway, neurotrophin and mTOR signaling (Figure 5D).

Five up-regulated miRNAs (miR-26a-5p, miR-129-1-3p, miR-186-5p, miR-23b-3p and miR-365-3p) were selected for further investigation since they were strikingly differentially expressed in MS-SCs-EVs when compared to SCs-EVs. qRT-PCR was performed to further validate the 5 most significantly altered miRNAs MS-SCs-EVs exhibited the highest increase in miR-23b-3p (14.61-fold), followed by miR-186-5p (9.26-fold), miR-129-1-3p (8.96-fold), miR-365-3p (8.68-fold) and miR-26a-5p (5.28-fold) (Figure 5F). We next predicted the target genes of those five miRNAs using bioinformatics tools (TargetScan and miRDB). Interestingly, neuropilin1was a common target of all five differentially expressed miRNAs (miR-23b-3p, miR-186-5p, miR-129-1-3p, miR-365-3p and miR-26a-5p) in MS-SCs-EVs (Figure 5E).

To determine the effect of these miRNAs on Nrp1, we constructed a luciferase vector containing wild-type or mutated 3'-UTR sequence of Nrp1 (Nrp1-3'-UTR-WT, Nrp1-3'-UTR-MUT) (Figure 5G). Of the five differentially expressed miRNAs, only miR-23b-3p could significantly decrease Nrp1-3'-UTR-WT luciferase activity (Figure 5H), but did not affect the luciferase activity of Nrp1-3'-UTR-MUT (Figure 5I). Therefore, we chose miR-23b-3p to further elucidate its functions on Nrp1 expression and neurite outgrowth.

### miR-23b-3p enhanced neurite outgrowth *in vitro* and* in vivo*

**Figure 6:**
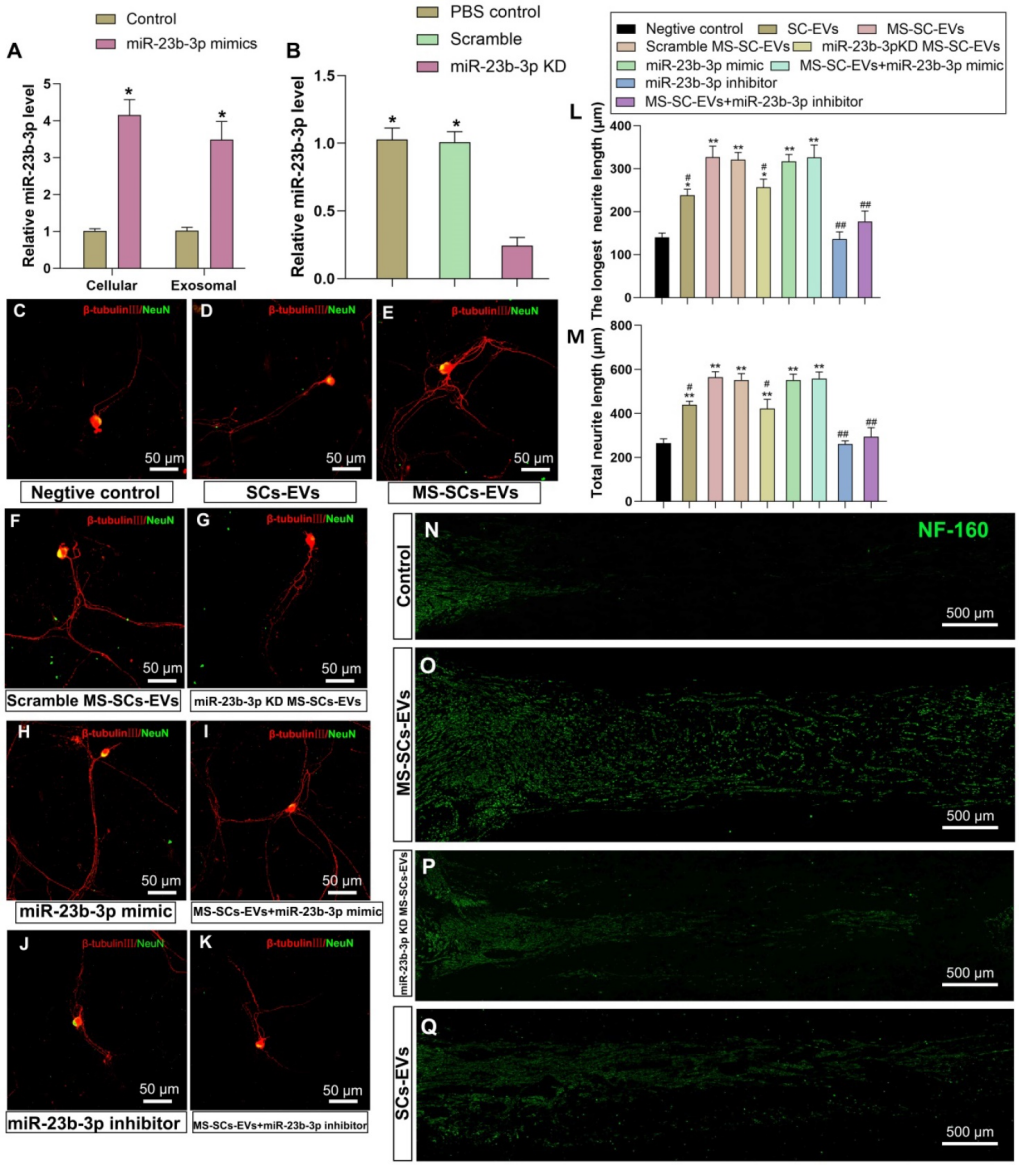
** EV-mediated transfer of miR-23b-3p enhances neurite outgrowth *in vitro* and *in vivo*.** (**A**) miR-23b-3p expression levels in EVs and corresponding cells of control group and miR-23b-3p mimic transfection group (n=5) (**B**) miR-23b-3p expression levels in EVs derived from MS-SCs, MS-SCs transfected with scramble plasmid and MS-SCs transfected with miR-23b-3p inhibitor measured by qRT-PCR (n=4). Representative images of DRG neurons stained with β-tubulin III (red) and NeuN (green) (**C**) DRG neurons transfected with the negative control, (**D**) DRG neurons treated with SCs-EVs, (**E**) DRG neurons treated with MS-SCs-EVs, (**F**) DRG neurons treated with scramble MS-SCs-EVs, (**G**) DRG neurons treated with miR-23b-3p KD MS-SCs-EVs, (**H**) DRG neurons transfected with miR-23b-3p mimics, (**I**) DRG neurons treated with MS-SCs-EVs + miR-23b-3p mimics, (**J**) DRG neurons transfected with miR-23b-3p inhibitors, (**K**) DRG neurons treated with MS-SCs-EVs+miR-23b-3p inhibitors. (**L-M**) Longest neurite length and total neurite length quantified and plotted for each group, as observed at 96 h after transfection. n=20 per group, scale bars: (C-K) 50 μm. Four weeks after surgery, representative images of regenerated axons stained for NF 160 (green) in the PBS control group (**N**), MS-SCs-EVs group (**O**), miR-23b-3p KD MS-SCs-EVs group (**P**), SCs-EVs group (**Q**); scale bars: (N-Q) 500 μm. The results are expressed as the mean ± SEM. Student's t-test and one-way ANOVA test with Tukey's post hoc test were used to examine the significance of results.* P<0.05 and ** P<0.01 for comparison with the negative control group or the miR-23b-3p KD group, #* P*<0.05 and ##* P*<0.01 for comparison with the MS-SCs-EVs group.

To determine the function of EV-derived miR-23b-3p, we knocked down its expression by transfecting SCs with lentivirus of antisense oligonucleotides or overexpressed it by using miR-23b-3p mimics. The overexpression by miR-23b-3p mimics resulted in a 4.71-fold and 4.11-fold increase in SCs and MS-SCs-EVs, respectively (Figure 6A). The knockdown of miR-23b-3p in SCs (miR-23b-3p KD) resulted in 79.8% decreased expression in MS-SCs-EVs (Figure 6B). The impact of miR-23b-3p on neurite outgrowth was further investigated in purified DRG neuron cultures (Figure 6C-K). The enhancement of MS-SCs-EVs on neurite outgrowth was significantly attenuated by miR-23b-3p KD (Figure 6E, G, L and M). Furthermore, miR-23b-3p mimics were sufficient to promote neurite outgrowth comparable to MS-SCs-EVs (Figure 6E, H, L and M). Further studies showed that simultaneous addition of MS-SCs-EVs and miR-23b-3p mimics did not elicit a synergistic effect on neurite outgrowth (Figure 6I, L and M). Moreover, miR-23b-3p inhibitor showed little effect on neurite outgrowth in DRG neuron cultures (Figure 6J, L and M), but significantly decreased the neurite outgrowth in neurons treated with MS-SCs-EVs (Figure 6K, L and M). Measurement of the length of the longest axons and the total length of neurites indicated that overexpression of miR-23b-3p and supplementation with MS-SCs-EVs containing miR-23b-3p significantly enhanced neurite outgrowth compared to the SCs-EVs group.

The effect of EV-derived miR-23b-3p on axonal regeneration was further investigated in a rat model of nerve injury. A 5-mm sciatic nerve defect was created in rats and was bridged with a PCL nerve conduit containing an equal volume of PBS, SCs-EVs, MS-SCs-EVs or miR-23b-3p KD MS-SCs-EVs. Four weeks after injury, the SCs-EVs group was found to enhance axonal regeneration compared to the PBS control group (Figure 6N and Q). The axonal regeneration was further enhanced by MS-SCs-EVs (Figure 6O), but was significantly inhibited by knockdown of miR-23b-3p in MS-SCs-EVs (Figure 6P). These observations suggested that the beneficial effect of MS-SCs-EVs on axonal regeneration was attributable, at least in part, to the elevated expression of miR-23b-3p in MS-SCs-EVs.

### EV-derived miR-23b-3p enhanced neurite outgrowth by targeting Nrp1

**Figure 7:**
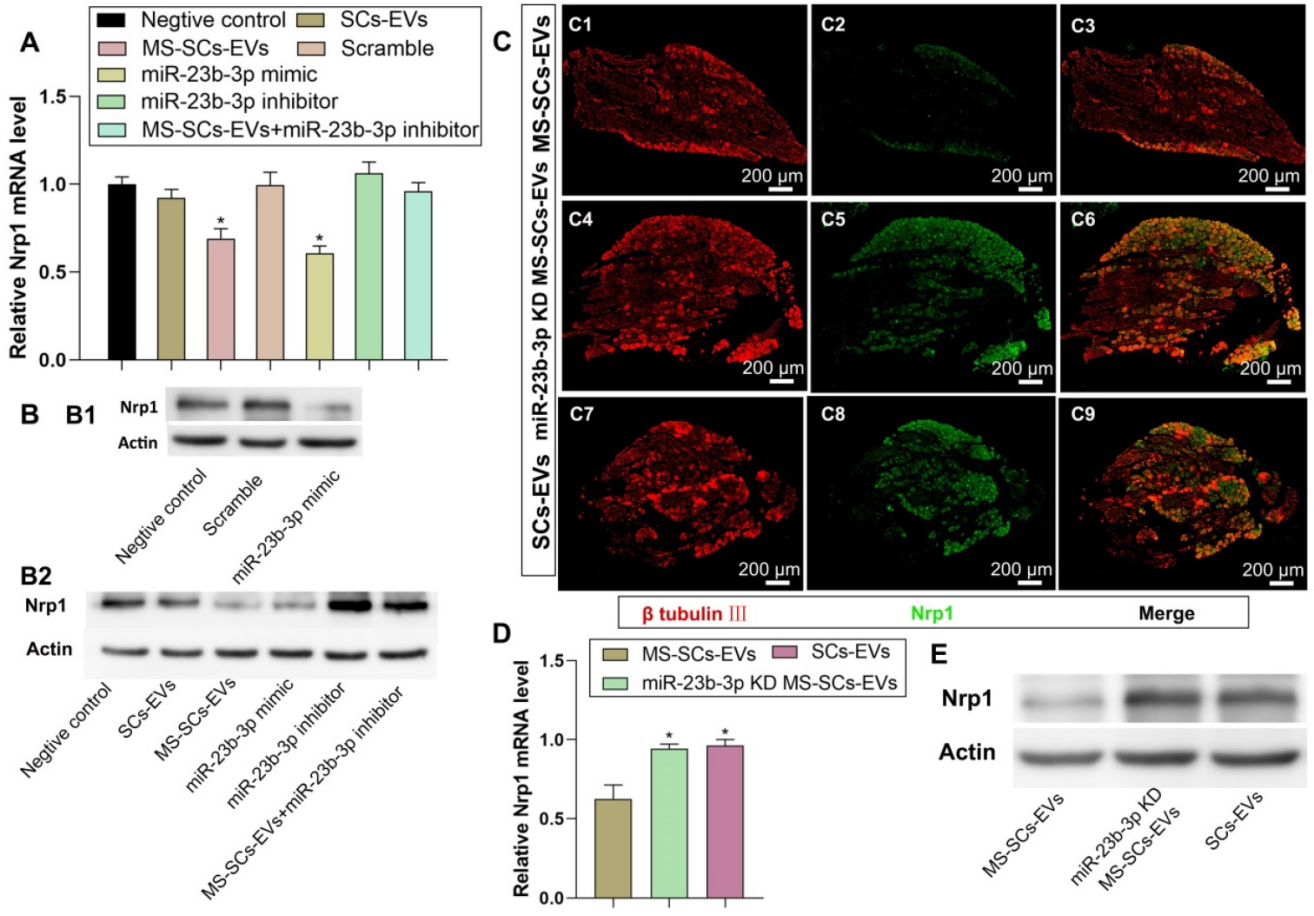
** Nrp1 is a direct target of miR-23b-3p in MS-SCs-EVs.** Notes: (**A**) Nrp1 mRNA levels in DRG neurons of each group measured by qRT-PCR (n=4). (**B**) Nrp1 protein expression in DRG neurons of each group (**C**) DRGs were stained for β-tubulin III (red) and Nrp1 (green) to demonstrate the Nrp1 protein expression 7 days after addition of MS-SCs-EVs (C1-C3), miR-23b-3p KD MS-SCs-EVs (C4-C6) and SCs-EVs (C7-C9) in the injury site of rats with sciatic nerve injury. n=6 per group, scale bars: (C) 200 μm. (**D**) Nrp1 mRNA levels (n=4) and (**E**) Nrp1 protein expression in DRGs 7 days after addition of MS-SCs-EVs, miR-23b-3p KD MS-SCs-EVs and SCs-EVs in the injury site of rats with sciatic nerve injury. The results are expressed as the mean ± SEM. One-way ANOVA test with Tukey's post hoc test were used to examine the significance of results.* P<0.05 for comparison with the negative control group or the MS-SCs-EVs group.

Nrp1 was a putative target gene of miR-23b-3p as it significantly decreased Nrp1-3'-UTR-WT luciferase activity. We investigated the effect of miR-23b-3p on endogenous Nrp1 expression in DRG neurons and detected significant attenuation of Nrp1 expression by MS-SCs-EVs or miR-23b-3p mimics (Figure 7A, B1, B2). Besides, the inhibitory effect of MS-SCs-EVs on Nrp1 expression was abolished by a miR-23b-3p inhibitor (Figure 7A, B2), suggesting the involvement of miR-23b-3p in the regulation of Nrp1 expression mediated by MS-SCs-EVs in neurons. To further confirm the effect of EV-derived miR-23b-3p on Nrp1 *in vivo*, a 5-mm sciatic nerve defect was generated in rats and was bridged with a PCL nerve conduit containing an equal volume of SCs-EVs, MS-SCs-EVs or miR-23b-3p KD MS-SCs-EVs (Figure 7C). L4-L6 DRGs were harvested 7 days after surgery and immunostained with β-tubulin Ⅲ and Nrp1. We observed that Nrp1 expression in DRGs was down-regulated in MS-SCs-EVs compared to the SCs-EVs group (Figure 7C1-C3, C7-C9). Further analysis showed that the Nrp1 expression in DRG neurons was elevated by knockdown of miR-23b-3p in MS-SCs-EVs (Figure 7C4-C6). These results were consistent with Nrp1 expression measured by Western blotting and qRT-PCR analysis (Figure 7D, E), confirming that Nrp1 is a direct target of MS-SCs-EVs derived miR-23b-3p.

**Figure 8:**
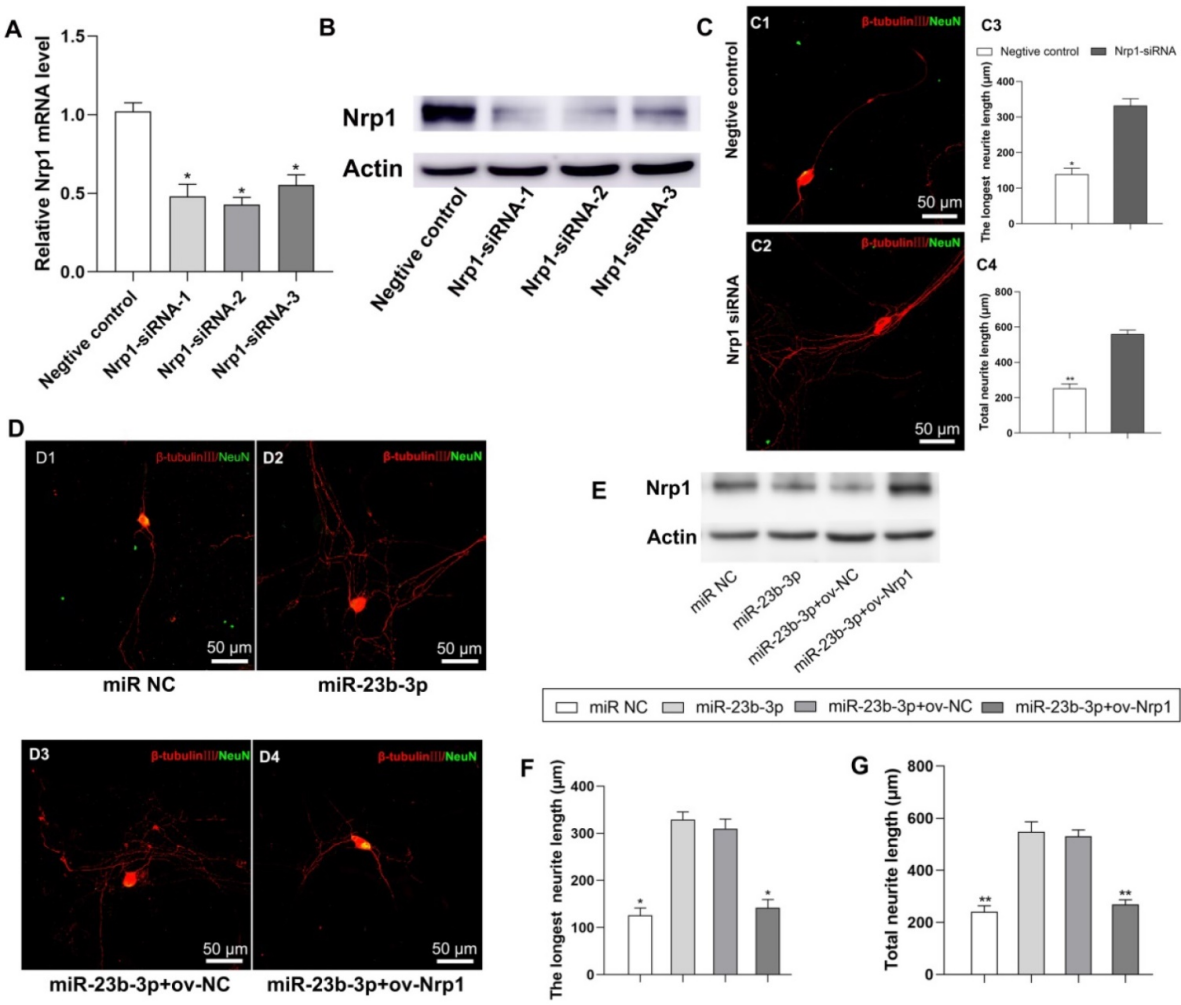
** miR-23b-3p enhances neurite outgrowth of DRG neurons by targeting Nrp1.** (**A**) Nrp1 mRNA levels in DRG neurons by qRT-PCR after transfection with 3 different siRNAs targeting Nrp1 or a negative control siRNA. (**B**) Nrp1 protein expression in DRG neurons after transfection with 3 different siRNAs targeting Nrp1 or a negative control siRNA. (**C**) Silencing of Nrp1 in DRG neurons significantly enhanced neurite outgrowth (n=20). (**D**) Nrp1 reintroduction into DRG neurons partially counteracted the enhancement of neurite outgrowth by miR-23b-3p. (**E**) miR-23b-3p down regulated Nrp1 protein expression in DRG neurons, while Nrp1 plasmid transfection restored Nrp1 protein expression in DRG neurons. (**F**) and (**G**) are the quantification of the longest neurite length and the total neurite length in each group, n = 20 per group. Scale bars: (C and D) 50 μm. The results are expressed as the mean ± SEM. Student's t-test and one-way ANOVA test with Tukey's post hoc test were used to examine the significance of results.* P<0.05, ** P<0.01 for comparison with the negative control group or the miR-23b-3p treated group.

Next, we examined the role of Nrp1in the beneficial effect of MS-SCs-EVs derived miR-23b-3p on neurite outgrowth. Three short interfering RNAs (siRNAs) were designed to knock down the expression of Nrp1 in neurons ((Figure 8A, B, *P*<0.05). Nrp1 siRNA-2 was used for further studies since it resulted in higher efficiency of Nrp1 silencing (Figure 8A, B, *P*<0.05), which significantly enhanced neurite outgrowth in DRG neurons (Figure 8C). We then restored Nrp1 expression by transiently transfecting DRG neurons with full-length Nrp1 plasmid (ov-Nrp1) or negative control vector (ov-NC) (Figure 8D). We found that miR-23b-3p down-regulated Nrp1 protein expression level, while Nrp1 plasmid restored the Nrp1 protein expression in DRG neurons (Figure 8E). The beneficial effects of miR-23b-3p on neurite outgrowth were partially offset by Nrp1 overexpression (Figure 8D, F, G). These results collectively suggested that MS-SCs enhanc neurite outgrowth through EV delivery of miR-23b-3p by targeting Nrp1in neurons.

## Discussion

In this study, we established an MS system to stress SCs *in vitro* and applied SPIONs to SCs to indirectly monitor their effect on neurons. We found that mechanical stimuli altered the cargo in MS-SCs-EVs and consequently modulated the intercellular communication between neurons and SCs.

The MS-SCs-EVs could be internalized by DRG neurons, and showed a stronger ability to enhance neurite outgrowth *in vitro* and nerve regeneration* in vivo* when compared to SCs-EVs. To identify the mechanism underlying the beneficial effect of MS-SCs-EVs on axonal regeneration, we performed comparative high throughput miRNA sequencing, and identified many differentially expressed miRNAs in MS-SCs-EVs. Functional studies demonstrated that miR-23b-3p showed a predominant role in MS-SCs-EVs, since depletion of miR-23b-3p abolished the MS-SC-EV-mediated enhancement on axonal elongation. Furthermore, we identified Nrp1 in neurons as the target gene of miR-23b-3p in MS-SCs-EVs. These findings collectively suggested that mechanical stimuli to SCs were able to promote axonal regeneration through SCs-derived EVs, which mediated the transfer of miR-23b-3p from mechanically-stimulated SCs to neurons and subsequently down-regulated Nrp1 expression in neurons.

It has been reported that several different nanoparticle materials can lead to reactive oxygen species (ROS) formation and actin cytoskeleton disruption, and many studies further demonstrated that nanoparticles increased ROS formation and reduced cell viability in a dose- and time-dependent manner 48. Studies have shown that endothelial cells incubated with 0.5 mg/mL iron oxide nanoparticles begin to show a statistically significant increase of ROS formation and cell length 49. Therefore, the concentration of added SPIONs is an important control factor for cytotoxicity. In our study, we examined the cytotoxicity of SPIONs for SCs by CCK-8 and flow cytometry, and 2 μg/mL of SPIONs was used in the present experiments. SPIONs also have been designed with a promising method as EV or exosome cargo together with therapeutic agents for effective targeting on the molecular level* via* an external MF 36. In their study, the citrate-coated very small iron oxide particles showed a small size of around 7 nm, which is suitable to be internalized by EVs or exosome. In our study, limited by the diameter of SPIONs we used, the SPIONs can hardly be internalized by the MS-SCs-EVs or SCs-EVs. SPIONs have been reported to induce degeneration of neurons in a dose dependent manner, thus particular caution has to be exercised when administering SPIONs that target the neuronal cells 50. Based on this consideration, we applied SPIONs to SCs and effect on neurons indirectly. To examine the effect of SPIONs on EVs biological function, we designed an experimental group of EVs derived from SCs only loaded with SPIONs (without MF). In addition, the NTA results showed that the yield of EVs derived from SCs and MS-SCs is (7.10±0.404) ×10^10^/flask and (7.27±0.504)×10^10^/flask, which demonstrated that mechanical stimulation had no effect on EV secretion of SCs.

The influence of mechanical stimuli on Schwann cell biology has attracted increasing attention since SCs are constantly subjected to mechanical strains from the movement of the limbs 11, 12. Accumulating evidence has shown that SCs are sensitive to mechanical stimuli and the stiffness of their environment 14-16. Chronic nerve compression in a murine model induced changes in SC myelin architecture, and resulted in increased proliferation in SCs without axonal injury 14. In addition, shear stress on SCs reduced the expression of myelin-associated glycoprotein and myelin basic protein in SCs *in vitro* 15, 16. Many studies have been performed to unveil the mechanobiology of SCs and identified several important regulators including extracellular matrix, cell adhesion molecules, cortical cytoskeleton, and YAP/TAZ signaling 13, 18. The studies focused on the direct effect of mechanical stimuli on SCs, but overlooked their potential impact on intercellular communication between SCs and neurons, which plays an essential role in the development and regeneration of the PNS. In the present study, the internalized SPIONs were observed by the SEM and TEM analysis, which is in line with the results of the quantification of intracellular irons. The combination of intracellular SPIONs and applied MF exerted synergic force upon SCs and caused the subsequent biological reactions. The total force acting upon a single SC can be calculated by the physical equations 35, 44. In a non-uniform magnetic field, the magnetic force ***F*** exerted on a SPION with magnetic moment*** m*** is shown as:

*F*= (*m***•**∇) *B* (1)

where ***B***is the magnetic flux density of the applied MF and ***m*** is the magnetic dipole.

The number of internalized SPIONs can be calculated from the corresponding internalized iron of SCs, and it was found 1.21±0.08 pg/cell corresponding to a number of particles per cell N^SPIONS/cell^~3.1×10^4^. A single SC will be thus subjected to a force ***F_cell_*** given by equation (1) multiplied for the number of internalized SPIONs:

*F_cell_*= *N^SPIONS/cell^F* (2)

Cell-to-cell communication is essential for physiological homeostasis, particularly in the nervous system 20, 51, and is based on paracrine factors, connexons, or direct cell-cell contact 52. EVs are recognized as a novel and promising paracrine mechanism for intercellular communication and play a significant role in the development and physiology of nervous systems 53, 54. Glia cells are an essential component of the nervous system, and EVs derived from microglia 55, oligodendrocytes 56, and astrocytes 57 have been shown to serve as modulators of intercellular interactions in the central nervous system (CNS). Exosomes derived from MSCs, SCs, and macrophages have also been shown to modulate nerve regeneration via EV-mediated transfer of informative cargoes 58. In the present study, we found that SCs-EVs were capable of enhancing axonal elongation, which is consistent with previous studies 24. More importantly, compared to SCs-EVs, MS-SCs-EVs showed a stronger ability to enhance axonal elongation*.* This finding is significant and highlights the therapeutic value of MS-SCs-EVs in the treatment of peripheral nerve injury. It was important to elucidate the underlying mechanism since it may provide new insights into the regulation of nerve regeneration in peripheral neuropathies.

Endosome-origin “exosomes” and plasma membrane-derived “ectosomes” (microparticles and microvesicles) assign an EV to a particular biogenesis pathway 59. According to the Minimal Information for Studies of Extracellular Vesicles guides line proposed by International Society for Extracellular Vesicles 59, the term “extracellular vesicle” should be used as a genetic term for all secreted vesicles, unless the EV is caught in the act of release by live imaging techniques which can trace its biogenesis pathway. In the present study, based on the isolation method of EVs in the present study, the majority of the obtained EVs are exosomes. Since we have no definitive tracing evidences, we prefer to use the term “extracellular vesicle” to describe the vesicles obtained in the present study. EVs can be secreted by almost all types of cells, are also found in multiple body fluids, and have a pivotal role in intercellular communication in physiological and pathological conditions 60. EVs transfer information *via* their cargoes in the nervous system, including DNAs, mRNAs, miRNAs, and proteins 57. A growing body of studies focus on the role of EV-derived miRNAs enriched in EVs that exert diverse functions in the nervous system 25. In the CNS, exosomal miRNA-132 has been found to mediate neural regulation of brain vascular integrity 61. Also, miRNAs transferred by exosomes from microglia and astrocytes could inhibit neuronal inflammation in scratch-injured neurons and modulate neuronal morphology and synaptic transmission 62, 63. In the PNS, an abundance of miRNAs was reported in axons and nerve terminals, likely transferred directly from SCs 64. Also, EVs originating from SCs, macrophages, and MSCs, have been shown to promote peripheral nerve regeneration, during which many miRNAs were critically involved 58.

In the current study, MS-SCs-EVs have been shown to promote axonal elongation, but the underlying mechanism was not clear. The function of EVs depends on their composition 65, and distinct compositions in different sources have been documented to have diverse functions 66. Most importantly, the tissue-specific environment was found to influence the miRNA content in adipose mesenchymal stem cells and bone marrow mesenchymal stem cells 67. For the mechanical load-bearing cells, mechanical stimuli were shown to influence the composition of EVs in MSCs and osteocytes 26, 28. Although SCs are known to be sensitive to mechanical strains, the effect of mechanical stimuli on the composition of SC-derived EVs is not clear.

Herein, we performed high-throughput miRNA sequencing to identify the miRNA cargoes in MS-SCs-EVs. Many miRNAs were differentially expressed in MS-SCs-EVs compared to SCs-EVs. We further characterized the function of the differentially expressed miRNAs derived from MS-SCs-EVs in axonal elongation. We identified a predominant role of miR-23b-3p in MS-SCs-EVs, since deprivation of miR-23b-3p in MS-SCs-EVs nearly abolished the MS-SCs-EV-mediated enhancement on neurite outgrowth and nerve regeneration. miR-23 is among the highly conserved vertebrate miRNAs that are involved in various biological processes 68. Studies have shown that adipose-derived exosomes deliver miR-23a/b and regulate tumor growth in hepatocellular cancer 69 and granuloma patients but not in lung adenocarcinoma patients with high expression level of miR-23b-3p in EVs 70. Also, plasma exosomal miR-23b-3p level has been reported to be down-regulated in Alzheimer's patients 71. Thus far, the role of miR-23b-3p in peripheral nerve injury is unknown. In this study, we directly transferred miR-23b-3p mimic to neurons, and found that it was capable of increasing the neurite length, suggesting a beneficial effect of miR-23b-3p on axonal elongation. However, the mechanism by which miR-23b-3p promotes axonal elongation remained unclear.

We next predicted the target genes of miR-23b-3p using bioinformatics tools (TargetScan and miRDB) and identified Nrp1 as the common target of five differentially expressed miRNAs (miR-23b-3p, miR129-1-3p, miR-186-5p, miR-26a-5p and miR365-3p) in MS-SCs-EVs. Neuropilin is a membrane-associated glycoprotein which can be classified into two subfamilies, including neuropilin 1 (Nrp1) and neuropilin 2 (Nrp2). Nrp1 is a receptor for Semaphorin 3A (Sema3A) and also acts as a co-receptor for vascular endothelial growth factor (VEGF) 72. Sema3A is an important axon repellent that can induce collapse and paralysis of spinal motor neuron growth cones and activation of Nrp1 results in axon retraction and impaired regeneration 73. Nrp1 together with its co-receptor plexinA triggers axonal retraction by destabilizing microtubules and microfilament networks. The collapsing response mediator protein class of microtubule-associated proteins serves as major signaling molecule in Nrp1-mediated axonal damage 74-76. Sema3A and Nrp1 are repulsive axon guidance proteins and elevated expression of Sema3A and Nrp1 after damage probably contributes to the failure of neuronal and axonal regeneration in the adult mammalian CNS 77, 78.What makes interpretation of Nrp1 regulation difficult is that it is not only a receptor for Sema3A but also a co-receptor for VEGF. The axon growth inhibitor Sema3A and the neurotrophic factor VEGF therefore are competing ligands of Nrp1 79, 80. It has reported that Nrp1 and VEGF-A are necessary for proper axonal wiring during brain development 81, Nrp1 has been shown to act directly in retinal ganglion neurons as an axon guidance receptor to promote commissural axon sorting and indirectly by regulating neurovascular co-patterning through its role in endothelial cell signaling. Furthermore, Nrp1 controls both axonal guidance and subcellular target recognition in basket cells. SEMA3A acts directly on Nrp1 during cerebellar development to shape their stereotyped axonal organization in basket cells 82. Chemorepulsive signaling via Sema3A and Nrp1 plays an essential role during motor axon path finding in the embryonal phase. Their inhibitory effect possibly prevents erroneous uncontrolled sprouting and connecting. There is compelling evidence for therapeutic potential of inhibitors of the Sema3A/Nrp1 pathway in the CNS and PNS. Galectin-1, which binds to Nrp1 and interrupts the Sema3A pathway led to ameliorated axonal regeneration and locomotor recovery after spinal cord injury in mice 83. In our study, suppression of Nrp1 in DRG neurons significantly improved axonal regrowth, as reported in a previous study 81. To determine the effect of differentially expressed miRNAs from EVs on Nrp1 expression in neurons, we constructed a luciferase vector containing wild-type or mutated 3'-UTR sequence of Nrp1. Of the five differentially expressed miRNAs, only miR-23b-3p significantly decreased Nrp1-3'-UTR-WT luciferase activity. Besides, delivering miR-23b-3p by MS-SCs-EVs to neurons inhibited Nrp1 expression in neurons. These findings suggested an inhibitory regulation of MS-SCs-EV-derived miR-23b-3p on Nrp1 expression in neurons, which might be responsible, at least in part, for the beneficial effect of MS-SCs-EVs on axonal regeneration.

In the present study, mechanical stimuli were found to have a significant influence on the composition of miRNA cargoes in SCs-derived EVs. Further analysis showed that transferring miR-23b-3p by MS-SCs-EVs to neurons decreased Nrp1 expression in neurons, which could help explain the beneficial effects of MS-SCs-EVs on axonal regeneration. A limitation of the current study is that we characterized only the miRNA profiles in MS-SCs-EVs. In the future, alterations in DNA, mRNA, proteins and other non-coding RNAs in the MS-SCs-EVs need to be examined. The findings of this study have both conceptual and therapeutic implications. The present findings reveal how SCs sense and transduce mechanical stimuli for EV-mediated intercellular communication between SCs and neurons, providing a better understanding of the mechanobiology of SCs. In addition, peripheral nerves are not only unique in their elasticity, but also have characteristic electrical properties. In our previous studies, electrical stimuli have been shown to exert a direct effect on SCs and neurons 84. Given the present findings of the mechanical stimuli-mediated communication between SCs and neurons, it is conceivable that electrical stimuli can enhance axonal regeneration by tuning the EV-mediated communication between SCs and neurons. This interesting possibility awaits further investigations. The present study also has therapeutic significance. EVs have been proven to be promising carriers for nucleic-acid-based therapeutics in recent years 85. EVs intrinsically express membrane-anchored and transmembrane proteins, which facilitate cellular uptake of encapsulated exosomal contents and promote tissue-directed transportation 86. Our study highlighted the therapeutic value of MS-SCs-EVs in enhancing peripheral nerve regeneration. MS-SCs-EVs exhibited superior effect in promoting nerve regeneration compared to SCs-EVs. Also, miR-23b-3p-enriched EVs might serve as an alternative to MS-SCs-EVs to promote peripheral nerve injury repair.

## Supplementary Material

Supplementary figures and tables.

## Figures and Tables

**Table 1:** Quantification of intercellular iron with different SPIONs concentrations

SPIONs concentration (μg/mL)	0.5	1	2	4	8
Quantification of intercellular iron (pg/cell)	0.28±0.03	0.49±0.05	1.21±0.08	1.94±0.13	2.12±0.19

SPIONs: superparamagnetic iron oxide nanoparticles.

**Table 2:** Most significantly altered miRNAs in MS-SCs-EVs compared to SCs-EVs

miRNA	Fold change (log2)	p value
**Up-regulated**		
rno-miR-23b-3p	3.7378	5.90E-08
rno-miR-186-5p	3.2582	1.65E-06
rno-miR-129-1-3p	3.1937	6.11E-06
rno-miR-365-3p	3.183	8.31E-06
rno-miR-26a-5p	2.4542	8.22E-05
rno-miR-9a-5p	2.4071	1.25E-04
rno-miR-455-3p	2.3855	1.66E-04
rno-miR-26b-5p	2.3201	1.97E-04
rno-miR-98-5p	2.266	2.93E-04
rno-miR-23a-3p	2.1666	3.71E-04
rno-miR-30c-5p	2.1512	4.13E-04
**Down-regulated**		
rno-miR-351-3p	-4.7875	2.22E-07
rno-miR-3473	-3.9132	1.38E-06
rno-miR-370-3p	-3.6888	3.92E-06
rno-miR-125a-3p	-3.2314	4.21E-05
rno-miR-494-3p	-3.1514	7.77E-05
rno-miR-214-3p	-3.0023	1.06E-04
rno-miR-423-5p	-2.806	2.75E-04
rno-miR-770-3p	-2.7896	4.18E-04
rno-miR-134-5p	-2.7373	4.61E-04

## Abbreviations

ASO: antisense oligonucleotides
CNS: central nervous system
DRG: dorsal root ganglion
EVs: extracellular vesicles
MF: magnetic field
MS-SCs-EVs: extracellular vesicles derived from Schwann cells loaded with magnetically mechanical stimulation
miR-23b-3p KD MS-SCs-EVs: extracellular vesicles derived from miR-23b-3p knocked down Schwann cells loaded with magnetically mechanical stimulation
NTA: nanoparticle tracking analysis
Nrp1: neuropilin 1
PNS: peripheral nervous system
SCs: Schwann cells
SCs-EVs: extracellular vesicles derived from Schwann cells
SEM: scanning electron microscopy
TEM: transmission electron microscopy
3'UTR: 3' untranslated region
