# Supplementary material for: Mechanical stimulation of Schwann cells promote peripheral nerve regeneration via extracellular vesicle-mediated transfer of microRNA 23b-3p
Source: Theranostics. 2020 Jul 11;10(20):8974–95. doi: 10.7150/thno.44912 (PMC7415818; doi:10.7150/thno.44912)
Supplement: Supplementary file 1 — Supplementary figures and tables. [file thnov10p8974s1.pdf]

Supplementary Figures

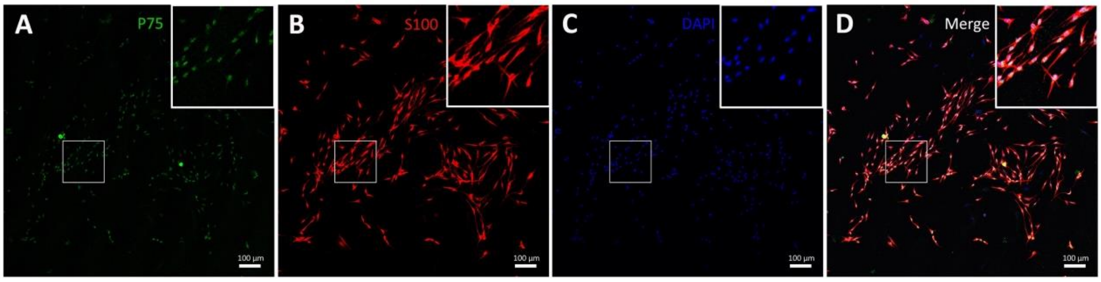

Figure S1: Characterization of primary SCs.

Double immunofluorescent staining showed the expression of p75 (A) and S100 (B) with DAPI nuclear counterstaining (C). Merge file (D).

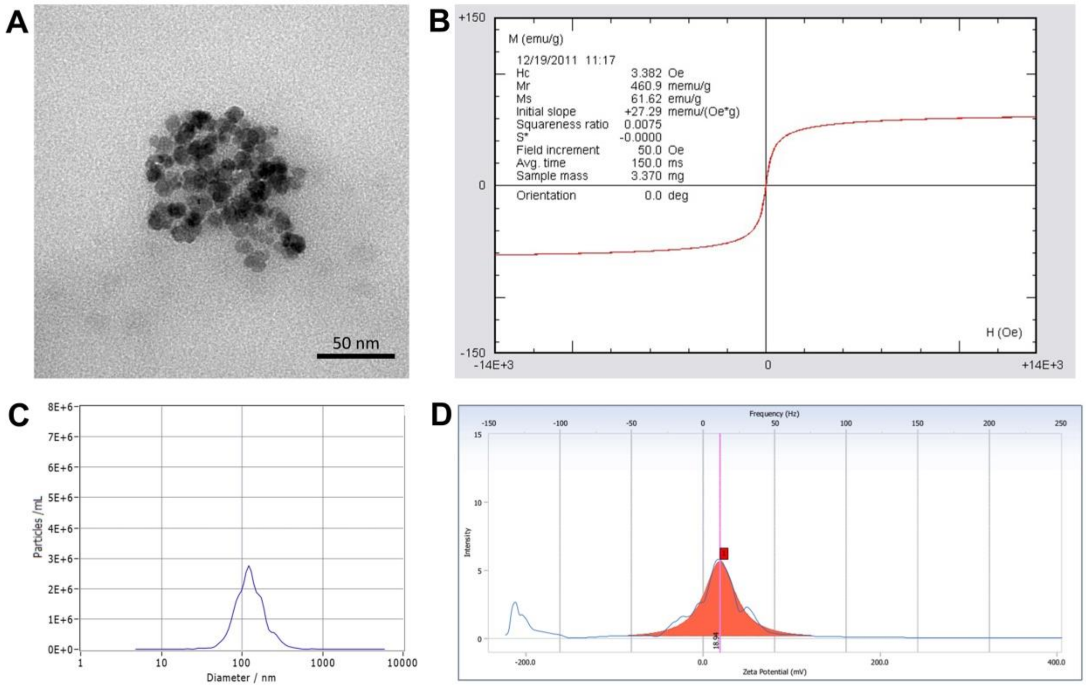

Figure S2: Characterization of SPIONs.

A: TEM analysis of SPIONs. B: Magnetization parameters of SPIONs. C: The overall distribution of SPIONs sizes. (D) The zeta potential of SPIONs.

Supplementary Table 1: siRNA sets used for knockdown Nrp1

siRNA	Location	Sense(5'-3')	Antisense(5'-3')
Nrp1-siRNA 1	Nrp1-rat-997	GCACCUACUGGAGUGAUAATT	UUAUCACUCCAGUAGGUGCTT
Nrp1-siRNA 2	Nrp1-rat-1566	GGGUGCCAUUUCCAAGGAATT	UUCCUUGGAAAUGGCACCCTT
Nrp1-siRNA 3	Nrp1-rat-322	CCUGGAGAACUAUAACUUUTT	AAAGUUAUAGUUCUCCAGGTT

Supplementary Table 2: Primer sets used for qRT-PCR

Primer	Sequence(5'-3')
Nrp1-Forward	GCGTGTCTTGCTGCACAAAT
Nrp1-Reverse	GGGTGCTCCCTGTTTCATCTAT
U6-Forward	CTCGCTTCGGCAGCACATATACT
U6- Reverse	ACGCTTCACGAATTTGCGTGTC
β-actin- Forward	TAGTTGCGTTACACCCTTTCTTG
β-actin- Reverse	TCACCTTCACCGTTCCAGTTT
